# HGF/c-Met Inhibition as Adjuvant Therapy Improves Outcomes in an Orthotopic Mouse Model of Pancreatic Cancer

**DOI:** 10.3390/cancers13112763

**Published:** 2021-06-02

**Authors:** Tony C. Y. Pang, Zhihong Xu, Alpha Raj Mekapogu, Srinivasa Pothula, Therese Becker, Susan Corley, Marc R. Wilkins, David Goldstein, Romano Pirola, Jeremy Wilson, Minoti Apte

**Affiliations:** 1 Pancreatic Research Group, South Western Sydney Clinical School, Faculty of Medicine and Health, Ingham Institute for Applied Medical Research, University of New South Wales, Sydney, NSW 2170, Australia; tony.pang@sydney.edu.au (T.C.Y.P.); zhihong.xu@unsw.edu.au (Z.X.); a.mekapogu@student.unsw.edu.au (A.R.M.); s.pothula@unsw.edu.au (S.P.); d.goldstein@unsw.edu.au (D.G.); rmpirola@ozemail.com.au (R.P.); js.wilson@unsw.edu.au (J.W.); 2 Surgical Innovations Unit, Westmead Hospital, Westmead, NSW 2145, Australia; 3 Westmead Clinical School, University of Sydney, Westmead, NSW 2145, Australia; 4 Centre for Circulating Tumour Cell Diagnostics and Research, Ingham Institute for Applied Medical Research, Sydney, NSW 2170, Australia; t.becker@unsw.edu.au; 5 Ramaciotti Centre for Genomics, School of Biotechnology and Biomolecular Science, University of New South Wales, Sydney, NSW 2052, Australia; s.corley@unsw.edu.au (S.C.); m.wilkins@unsw.edu.au (M.R.W.)

**Keywords:** pancreatic cancer, metastasis, circulating rare cells, circulating pancreatic stellate cells, circulating tumour cells, circulating stromal cells, cancer−stromal interactions, pancreatic stellate cells, hepatocyte growth factor, adjuvant treatment, cancer-associated fibroblasts

## Abstract

**Simple Summary:**

Pancreatic cancer (PC) has a poor prognosis. Even though surgical resection and adjuvant chemotherapy is the most effective therapy, recurrence remains common. In this paper, we investigate the effectiveness of dual inhibition of hepatocyte growth factor (HGF) and c-MET when used as treatment after surgical resection of PC in mice. The HGF/c-Met pathway is a major mediator of pancreatic stellate cell (stromal cell)—PC cell interactions. Using single-cell RNA sequencing, we also investigated the existence of co-metastasising cells, circulating pancreatic stellate cells (cPSCs), as facilitators of PC metastasis. We found that HGF/c-Met inhibition reduced both the risk and rate of disease progression after resection and that this effect was associated with reduced cPSC counts. In conclusion, this study is the first to demonstrate the efficacy of adjuvant HGF/c-Met inhibition and is also the first to confirm the existence of cPSCs in PC.

**Abstract:**

Background: Inhibition of hepatocyte growth factor (HGF)/c-MET pathway, a major mediator of pancreatic stellate cell (PSC)−PC cell interactions, retards local and distant cancer progression. This study examines the use of this treatment in preventing PC progression after resection. We further investigate the postulated existence of circulating PSCs (cPSCs) as a mediator of metastatic PC. Methods: Two orthotopic PC mouse models, produced by implantation of a mixture of luciferase-tagged human pancreatic cancer cells (AsPC-1), and human PSCs were used. Model 1 mice underwent distal pancreatectomy 3-weeks post-implantation (*n* = 62). One-week post-resection, mice were randomised to four treatments of 8 weeks: (i) IgG, (ii) gemcitabine (G), (iii) HGF/c-MET inhibition (HiCi) and (iv) HiCi + G. Tumour burden was assessed longitudinally by bioluminescence. Circulating tumour cells and cPSCs were enriched by filtration. Tumours of Model 2 mice progressed for 8 weeks prior to the collection of primary tumour, metastases and blood for single-cell RNA-sequencing (scRNA-seq). Results: HiCi treatments: (1) reduced both the risk and rate of disease progression after resection; (2) demonstrated an anti-angiogenic effect on immunohistochemistry; (3) reduced cPSC counts. cPSCs were identified using immunocytochemistry (α-smooth muscle actin+, pan-cytokeratin−, CD45−), and by specific PSC markers. scRNA-seq confirmed the existence of cPSCs and identified potential genes associated with development into cPSCs. Conclusions: This study is the first to demonstrate the efficacy of adjuvant HGF/c-Met inhibition for PC and provides the first confirmation of the existence of circulating PSCs.

## 1. Introduction

Pancreatic cancer (pancreatic adenocarcinoma, PC) is a poor-prognosis cancer, with an overall 5-year survival rate of around 9% [1,2]. In patients with non-metastatic PC, surgical resection combined with modern systemic chemotherapy therapy remains the only potentially curative treatment modality [3]. Unfortunately, recurrence of disease, even for patients who undergo resection and achieve macroscopic clearance of the tumour, is common. Indeed, in patients who do not receive adjuvant systemic therapy, median disease-free survival after surgery is reported to be less than 7 months [4]. 

Consequently, post-resection (adjuvant) therapy is currently recommended for all patients undergoing surgery, regardless of primary tumour staging [3,5]. However, recurrence rates with current adjuvant therapies remain high, indicating a critical need for more effective systemic treatment. 

Interactions between cancer cells and pancreatic stellate cells (PSCs, which produce the collagenous stroma of cancer) are now acknowledged as key drivers of cancer progression. One of the important signalling pathways that mediates cancer cell–PSC interactions is the hepatocyte growth factor (HGF)/c-Met pathway [6,7,8]. Using an orthotopic model of pancreatic cancer, wherein tumours were produced by implantation of a mixture of cancer cells and pancreatic stellate cells, we previously demonstrated that inhibition of both the ligand HGF and its receptor c-MET in combination with gemcitabine (cytotoxic agent) results in the virtual elimination of metastasis in both early and advanced-stage unresected pancreatic cancer [8,9,10]. Our hypothesis is that this triple therapy approach to inhibit metastases is also effective in the post-resection (adjuvant) setting, given that metastatic disease is a major cause of recurrence after curative resection [4,11]. 

Using our orthotopic model of pancreatic cancer noted above, we have also previously reported that PSCs not only facilitate local tumour growth but can move from the primary tumour to distant metastatic sites, where they likely facilitate cancer cell seeding and growth [12]. As a logical extension of this observation, we have postulated the existence of circulating PSCs (cPSCs), analogous to circulating tumour cells (CTCs) [13]. 

The aims of this study were (i) to assess the effects of adjuvant treatment (HGF/c-Met inhibition, with or without gemcitabine) on disease progression and on circulating cells (tumour cells and PSCs) in a post-resectional orthotopic model of pancreatic cancer and (ii) to use single-cell RNA-sequencing (scRNA-seq) to characterise circulating PSCs in the metastatic setting of an unresected model of pancreatic cancer.

## 2. Materials and Methods

A more detailed description of the methods is provided in Appendix A.

### 2.1. Orthotopic Mouse Models

**Figure A1:**
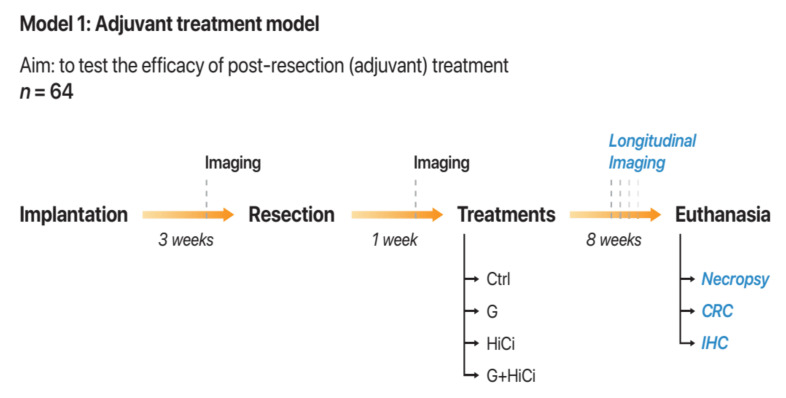
Flow diagram summarising the adjuvant treatment mouse model (Model 1). The aim was to test the efficacy of HGF/c-Met inhibition therapy (HiCi, rilotumumab and com-pound A) with or without gemcitabine (G) as adjuvant treatments for PC. The drugs were given in a 2 × 2 factorial fashion. Bioluminescence imaging was performed both pre- and post-resection. The major outcome measures (highlighted in blue) included longitudinal measures of tumour burden (as assessed by in vivo bioluminescence imaging), recurrence at necropsy (both macroscopic and microscopic), circulating rare cell numbers and immuno-histochemical assessment of recurrent tumours. Abbreviations: CRC, circulating rare cells. IHC, immunohistochemistry.

**Figure A2:**
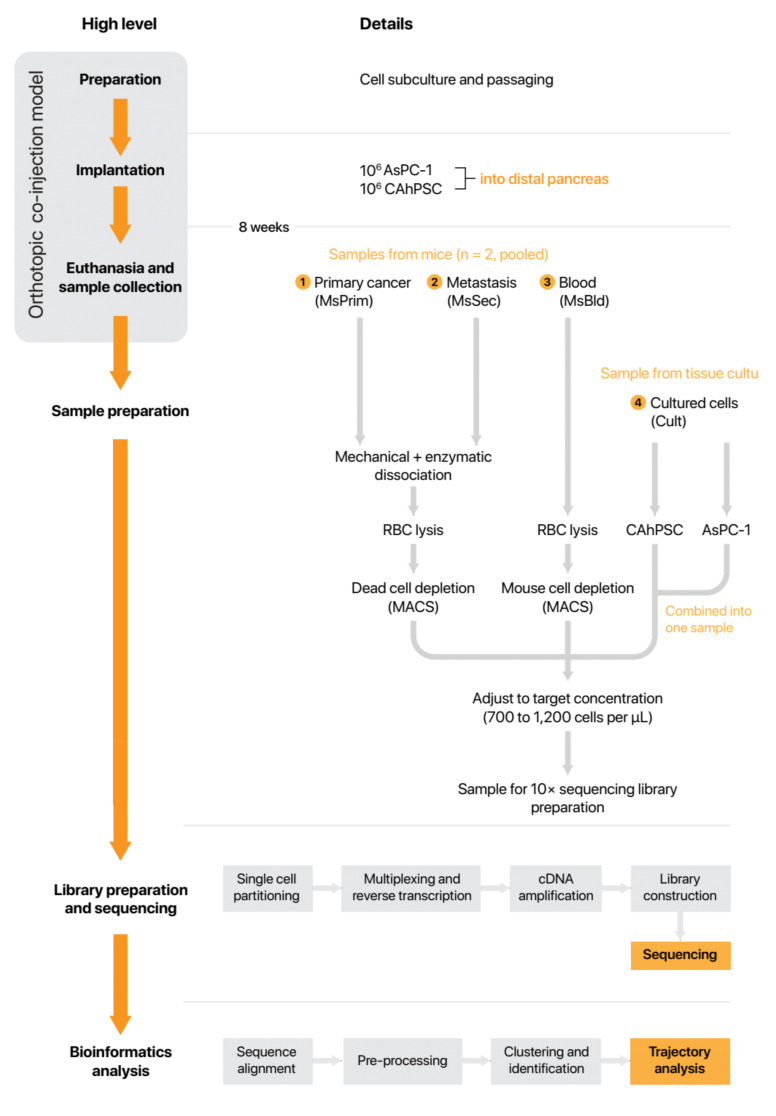
Flowchart summarising Model 2 (single cell RNA characterisation). Four samples were analysed. Three of these samples were derived from *n* = 2 mice: primary cancer (MsPrim), metastatic (secondary) cancer deposits (MsSec) and portal vein blood (MsBlood). The fourth sample was of cultured cancer associated human PSCs (CAhPSC) and AsPC-1 pancreatic cancer cells. This latter sample was used as a reference during analysis of mouse-derived cells. Tumours were dissociated into a single cell suspension and under-went dead cell depletion with magnetic cell separation (MACS), while circulating tumour cells and circulating pancreatic stellate cells were enriched using mouse-cell depletion. Abbreviations: CAhPSC, cancer-associated human pancreatic stellate cells. MACS, magnetic cell separation. RBC, red blood cells.

Two orthotopic mouse models were used in this study: Model 1 (adjuvant treatment model) and Model 2 (cPSC characterisation model). The aim of Model 1 was to test the effectiveness of HGF/c-Met inhibition treatment in the post-resection setting. This model thus involved tumour implantation, tumour resection, drug treatments and disease analysis (tumour burden, circulating cells, immunohistochemistry). Model 2 was an observational model designed to identify and characterise circulating PSCs by single-cell RNA-sequencing (scRNA-seq). Thus, this model involved only tumour implantation, the passage of time for tumour progression and then collection of blood and tissue for scRNA-seq analysis. Summary flowcharts for each model are shown in Figure A1 and Figure A2. 

### 2.2. Tumour Implantation

The basic schema for these mouse models has been described previously [14,15]. Briefly, athymic nude mice (BALB/c-*Fox1*^nu^/Ausb), aged 8−10 weeks and weighing 16−18 g, were injected in the distal pancreas with a mixture of 10^6^ luciferase-tagged AsPC-1 cells (kindly provided by Professor Takashi Murakami, Saitama University, Saitama, Japan) and 10^6^ cancer-associated human PSCs (Pancreatic Research Group PSC bank) in 50 μL of phosphate-buffered saline (PBS). Humane endpoints included loss of body weight > 20%, features of untreatable distress and tumour size > 1 cm^3^. Mortality from tumour progression was not considered as an acceptable humane endpoint; therefore, no survival analysis was possible.

### 2.3. Tumour Resection

Tumour resection was only performed in the adjuvant treatment model (Model 1). The period between implantation and resection was 3 weeks (determined by empiric optimisation). Bioluminescence imaging, described below, was performed the day prior to planned resection to exclude mice with clearly disseminated disease. 

**Figure A3:**
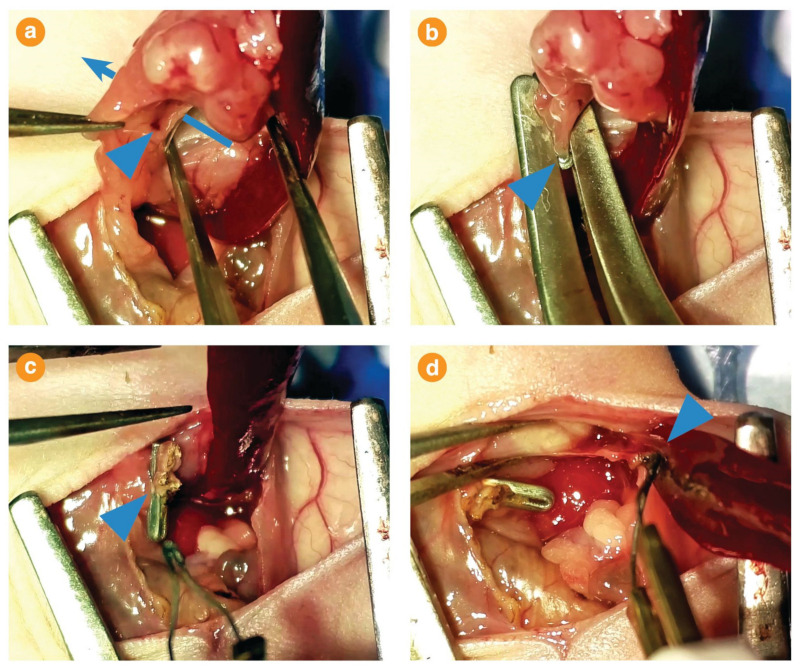
Distal pancreatectomy and splenectomy. These photographs were taken during the procedure and illustrates the key steps. (**a**) A “window” dorsal to body of the pancreas is dissected bluntly (Arrow: passes through the window. Arrowhead: lienopancreatic vein). (**b**) A titanium ligation clip (arrowhead) is applied on the pancreatic body proximal to the tumour. (**c**) The pancreas is transected with cautery (arrowhead: cauterised cut-surface of the pancreas, with adjacent titanium ligation clip). (**d**) Lienogastric vessels (arrowhead) is cauterised at the cranial pole of the spleen.

The tumour resection procedure has been described previously [15]. Briefly, the mouse was anaesthetised, the suture line excised and distal pancreatectomy/splenectomy performed. The pancreatic transection margin was sealed with a titanium ligation clip. The entire procedure is summarised in Figure A3.

### 2.4. Treatments

In Model 1, mice were treated with gemcitabine and/or combination rilotumumab and Compound A (c-Met small molecule inhibitor) in a 2 × 2 factorial design. The four groups (*n* = 8 mice/group) were as follows:

- **Control** (**Ctrl**)**:** IgG, 300 μg/mouse, by intraperitoneal (i.p.) injection daily (Amgen Inc., Thousand Oaks, CA, USA) and soybean oil, 10 μL/g by daily oral gavage (Sigma-Aldrich Pty Ltd., Castle Hill, NSW, Australia);
- **Gemcitabine** (**G**)**:** Gemcitabine, 75 mg/kg i.p. daily (Hospira, Mulgrave, VIC, Australia) alone;
- **HGF/c-Met inhibition** (**HCI**)**:** combination of rilotumumab, 300 μg/mouse i.p. daily (Amgen Inc.) and Compound A, suspended in soybean oil, 60 mg/kg by daily oral gavage (Amgen Inc.);
- **Combination of Gemcitabine and HGF/c-Met inhibition (G+HiCi)**: Gemcitabine, rilotumumab and Compound A.

Treatments were commenced 1-week post-resection and continued for 8 weeks (Figure A1).

### 2.5. Assessment of Effects of Treatment

For the adjuvant treatment model (Model 1), bioluminescence imaging was performed weekly after the implantation of the tumour until the end of the treatment period, so as to non-invasively track tumour progression. D-luciferin (15 mg/mL. PerkinElmer Inc., Waltham, MA, USA) was administered by intraperitoneal injection and imaging was performed using IVIS Lumina II (Caliper Life Sciences, Hopkinton, MA, USA), 18−26 min after injection. Image data analysis was performed using Living Image version 4.5.5 (64-bit) for Windows (Caliper Life Sciences). Total tumour burden was semi-quantitatively estimated by the ventral bioluminescent flux of the mouse.

The final tumour burden was assessed at necropsy. The abdominal and chest cavities were then examined for evidence of metastasis, which was recorded on a standard chart. The primary tumour was excised and the tumour volume estimated according the formula (½ × length × width × height) [16].

For Model 2, primary and metastatic nodules were excised at necropsy. These samples were processed separately into single-cell suspensions using enzymatic and physical dissociation using the Tumour Dissociation Kit and GentleMACS Octo (Miltenyi Biotec, Macquarie Park, NSW, Australia) as per the manufacturer’s instructions. Dead cells were removed from the resulting single-cell suspension by the use of the Dead Cell Removal Kit and the MACS magnetic cell separator system (Miltenyi Biotec, Macquarie Park, NSW, Australia)

### 2.6. Circulating Tumour Cells and Circulating Pancreatic Stellate Cells

At necropsy, 20 mL/kg of blood was collected from the portal vein and immediately placed into a CellSave tube (for Model 1; Menarini-Silicon Biosystems) or an EDTA tube (for Model 2; Interpath Service Pty Ltd., West Heidelberg, Victoria, Australia).

Circulating cell enrichment in Model 1 was performed using a filtration-based system, CellSieve platform (Creatv Micro Tech, Rockville, MD, USA), according to the manufacturer’s instructions. Circulating cell enrichment in Model 2 was performed using a negative immunomagnetic technique using the AutoMACS Pro cell separator and Mouse Cell Depletion Microbead cocktail kit (Miltenyi Biotec, Macquarie Park, NSW, Australia), as per the manufacturer’s instructions. 

### 2.7. Immunocytochemistry

Circulating tumour cells and circulating stellate cells were fixed with 4% paraformaldehyde, permeabilised with 0.3% Triton x-100 (Sigma-Aldrich Pty Ltd., Castle Hill, NSW, Australia) and blocked with normal donkey serum (Jackson ImmunoResearch Laboratories Inc., West Grove, PA, USA). They were then incubated with rabbit polyclonal anti-CD45 primary antibody (1:200. Abcam plc, Cambridge, UK), and donkey anti-rabbit IgG conjugated with AlexaFluor 647 (1:800. Jackson ImmunoResearch Laboratoraties Inc.). After further blocking with normal mouse serum (Jackson ImmunoResearch Laboratories Inc.), the cells were incubated with monoclonal mouse anti-human α-smooth muscle actin (clone 1A4) antibody conjugated with Cyanine 3 (1:150. Sigma-Aldrich Pty Ltd.) and monoclonal mouse anti-human pan-cytokeratin (clone C-11) antibody conjugated with fluorescein isothiocyanate (FITC) (1:450. Sigma-Aldrich Pty Ltd.). This was mounted and nuclear stained with Fluoromount-G with DAPI (4′,6-diamidino-2-phenylindole) (Life Technologies Corporation, Tullamarine, VIC, Australia).

Since α-SMA is also expressed by other mesenchymal cells such as fibroblasts, the identity of true cPSCs was confirmed by chemically quenching selected filtration membranes and re-staining the membranes for PSC-specific markers, desmin and glial fibrillary acid protein (GFAP). Quenching of fluorescent signal (in preparation for re-staining) was performed chemically with sodium borohydride (1 mg/mL. Sigma-Aldrich Pty Ltd., Castle Hill, NSW, Australia) based on the method of Adams et al. [17]. Re-staining was then performed by 5% bovine serum albumin (Roche Diagnostics, Indianapolis, IN, USA), followed by incubation with monoclonal mouse anti-human glial fibrillary acidic protein (clone GA5, conjugated with Alexafluor 488) (1:100. Novus Biologicals USA, Centennial, CO, USA) and monoclonal rabbit anti-human desmin (clone Y66, conjugated with Alexafluor 594) (1:100, Abcam plc, Cambridge, UK). Mounting was again with Fluoromount-G with DAPI.

**Figure A4:**
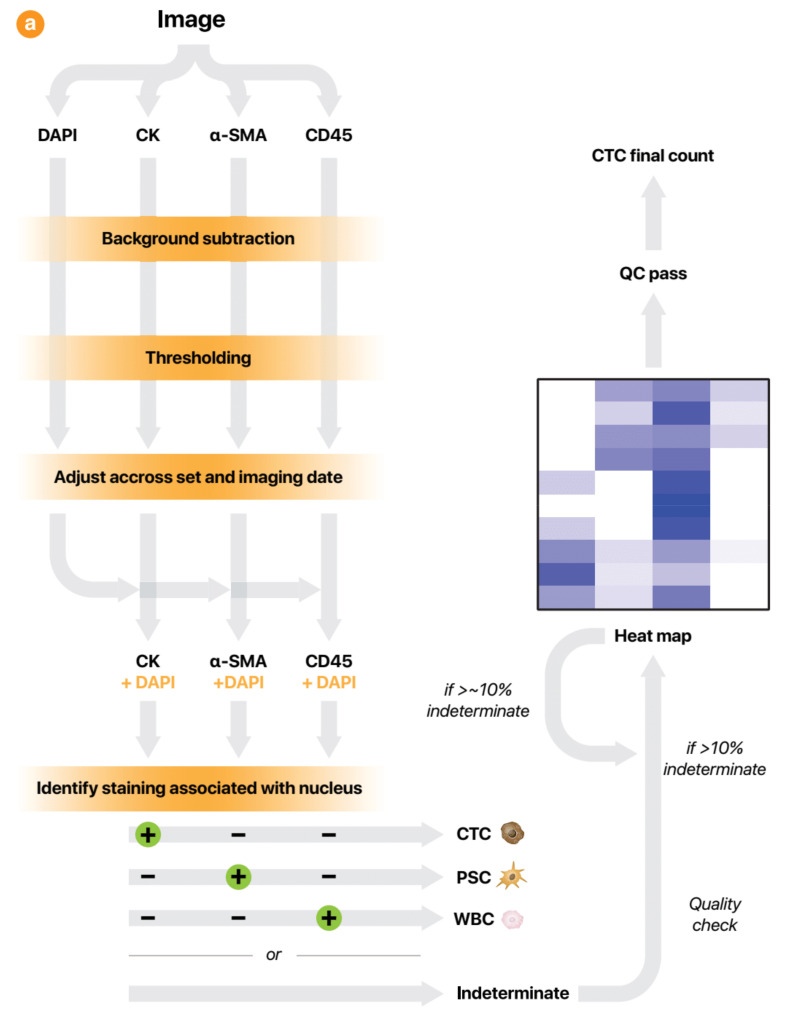

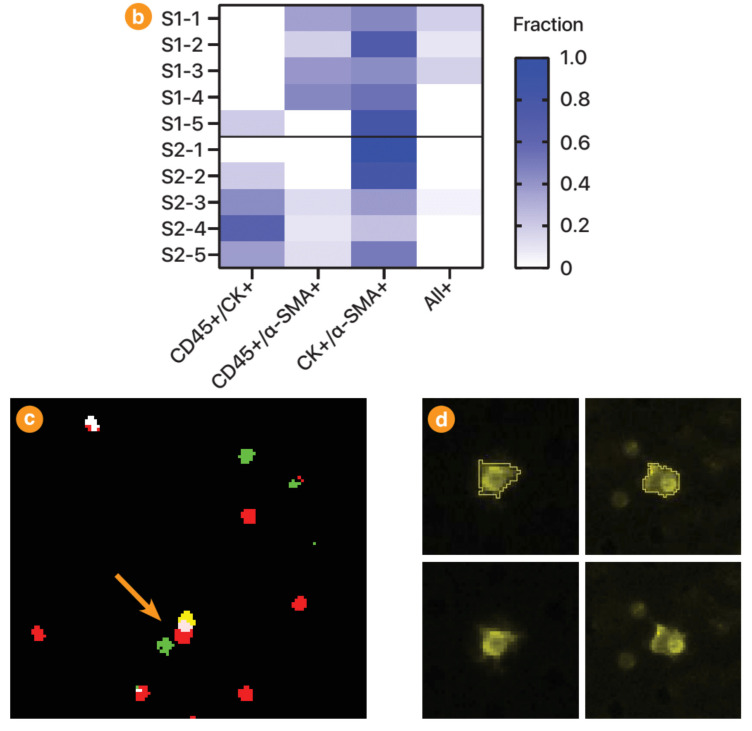
Summary of the semi-automated counting strategy. (**a**) Images were processed as four separate channels for background subtraction and initial thresholding. In this process, the experimenter was blinded to the treatment groups but not to the details regarding animal set and imaging date. These factors were taken into account to minimise the variation between samples of the same set and those which were imaged on the same day. After combining the images, a quality check (QC) was performed by screening for overlapping (indeterminate) staining. The thresholding for samples with >10% indeterminate cells were adjusted, aided by the pattern of indeterminate staining as visualised by a heatmap. (**b**) Example of the QC heatmap, which provides guidance for further threshold adjustments. Each row represents a low powered field (LPF). Each sample comprised five LPFs. Each column represents the staining characteristics as determined by the current threshold settings. The colour of the cells represent the fraction of the total number of indeterminate cells in the LPF with the given staining characteristic. In this heatmap, sample 1 (S1) demonstrates a high fraction of indeterminate cells with CD45+/α-SMA+ and CK+/α-SMA+, indicating that the α-SMA threshold is likely too low. In contrast, sample 2 (S2) has a high proportion of indeterminate cells with CD45+/CK+ and CK+/α-SMA+. This indicates that the CK threshold required adjustment. (**c**) Example of a binary image map used to identify CTC clusters. The cell identity of each cell is represented by a colour (leucocyte: red, CTC: green, cPSC: yellow and indeterminate: white). In this image, most cells exist as individual cells (five leucocytes, two CTCs, one indeterminate), except for a cell cluster composed of a cPSC, leucocyte and a CTC (indicated by the arrow). The cluster represented in this binary image map corresponds to the third heterotypic cluster shown in Figure 1d. (**d**) Examples of using the *magic wand* function to mark out a region of interest (ROI) corresponding to cPSCs. Once a ROI was determined, the measurement tool in ImageJ was used to measure characteristics such as maximum Feret diameter and staining intensity.

Cells were enumerated using a semi-automated technique using ImageJ version 1.52i (National Institutes of Health, Bethesda, MD, USA). The process is summarised in Figure A4.

### 2.8. Immunohistochemistry

Immunohistochemistry of paraffin-embedded sections of local recurrent tumour of the adjuvant treatment model (Model 1) was performed in standard fashion. Primary antibodies used included rabbit polyclonal anti-DCLK1 (1:800. Sigma-Aldrich Pty Ltd., Castle Hill, NSW, Australia), mouse monoclonal anti-human cytokeratin (clones AE1/AE3) (1:100. Agilent Technologies Pathology, Mulgrave, VIC, Australia), rabbit polyclonal anti-Ki67 (1:300. Abcam plc, Cambridge, UK) and rabbit polyclonal anti-CD31 (1:50. Abcam plc). This was followed by incubation with relevant secondary antibodies—Goat anti-rabbit immunoglobulin (Agilent Technologies Pathology) and goat anti-mouse immunoglobulin (Agilent Technologies Pathology)—and DAB visualisation. TUNEL (terminal deoxynucleotidyl transferase dUTP nick-end labelling) staining was performed using an in situ cell death detection kit (Roche Diagnostics, Indianapolis, IN, USA). Morphometric analysis and cell counting was performed using ImageJ version 1.52i.

### 2.9. Single-Cell RNA Sequencing

This was performed only for Model 2. Cell partitioning was performed using the 10x Genomics Chromium platform (10x Genomics, Pleasanton, CA, USA) according to the manufacturer’s instructions. Sequencing was performed at the Ramaciotti Centre for Genomics (UNSW) using the Illumina NovaSeq 6000 platform (Illumina, Inc., San Diego, CA, USA) with the SP 100 cycle flow cell with read lengths 28, 8 and 91 bp.

### 2.10. Bioinformatics

Generation of expression matrices was performed using the CellRanger pipeline, version 3.1.0 (10× Genomics). A combined reference genome was based on GRCh38 human assembly (Ensemble release 93), GRCm38 mouse assembly (release 93), modified firefly (*Photinus pyralis*) luciferase transgene, *luc+*, from the pGL3 Basic plasmid (Promega Corporation, Madison, WI, USA). The basic sequencing and alignment metrics are shown in Table A1.

Processing of the expression matrix data was performed using Seurat (version 3.1.4) [18] and Monocle (version 2.14.0) [19] packages in R (version 3.6.2). Cell filtering, gene expression normalisation, scaling and centering were performed. Linear and non-linear dimensional reduction was performed and visualised by principal component analysis and UMAP/t-SNE (uniform manifold approximation and projection/t-stochastic neighbour embedding) plots, respectively. 

Circulating pancreatic stellate cells were identified using three different methods, although the broad strategy involved first distinguishing human (xenografted) from mouse (native) cells, followed by distinguishing PSCs from cancer cells (See Section 3.2.1 or Appendix A.11.1). Single-cell trajectories were constructed using the reversed graph embedding technique implemented by Monocle 2 [19]. A more detailed description of the bioinformatics techniques used may be found in the Appendix A.

### 2.11. Non-Bioinformatics Statistical Analysis

Descriptive statistics for parametric, non-parametric and categorical variables were presented as mean ± SE, median (interquartile range, IQR) and *n* (%), respectively. Raw circulating rare cell counts were adjusted to account for the sample collection volume and reported as counts per 200 μL of sampled blood. Basic inferential tests, including ANOVA/*t*-test, Kruskall–Wallis test and Fisher’s exact/chi-squared tests, were first performed to explore the relationship between variables. If the global test demonstrated a near-significant result (*p*-value < 0.1), then post-hoc Tukey’s multiple comparison test was performed.

For regression modelling, treatments were considered as two independent treatment groups due to the 2 × 2 factorial pattern. First-order interactions were included in initial regression models, but these were dropped if the corresponding *p*-value exceeded 0.1 or if only one of the treatments was significant. Other independent variables were included in the model if these were found to be significant or near-significant (*p* < 0.15) on univariate analysis. Regression models used included multilevel mixed-effects models (to account for animal *set* level clustering and for longitudinal analysis of tumour burden), Poisson regression (with robust standard errors) (for CTCs and cPSC counts) and logistic regression analysis (for probability of progressive disease). Further details of these statistical models are described in the Appendix A.

All non-bioinformatics statistical analysis and data visualisation were performed using either GraphPad Prism version 8.3.0 for Windows (GraphPad Software LLC, San Diego, CA, USA) or Stata 15.1 IC for Windows (StataCorp LLC, College Station, TX, USA). 

## 3. Results

The 64 mice that underwent resection with macroscopically clear margins were randomised to treatments. Two mice died early and received less than 1 week of drug treatment. As this study aims to evaluate the anti-tumoural effects of treatments, these two mice were excluded from further analysis. The baseline characteristics of these mice were not significantly different amongst the four treatment groups (Table 1).

### 3.1. Effects of HGF/c-Met Adjuvant Treatments

#### 3.1.1. HGF/c-Met Inhibition Inhibited Tumour Progression Post-Resection

**Figure 1:**
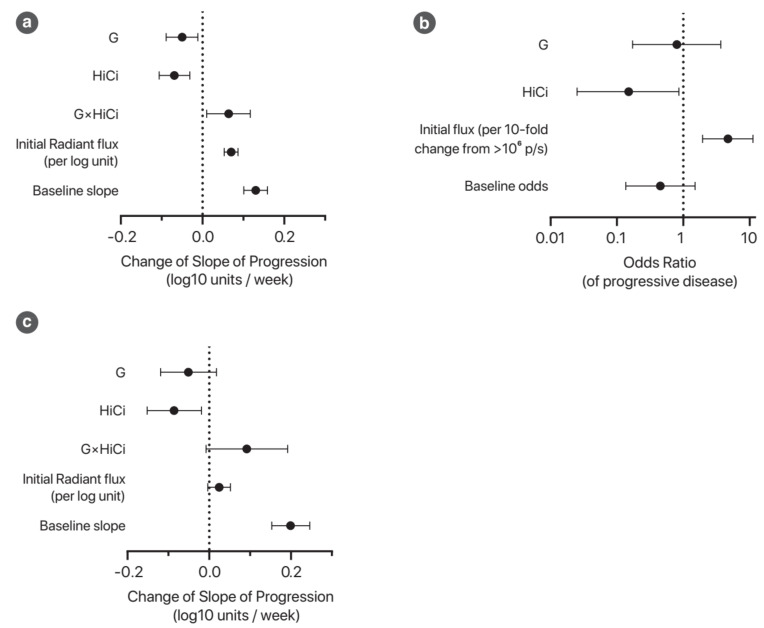
Progression of disease with adjuvant treatment (*n* = 62). (**a**) Graphical representation of the mixed-effects model for longitudinal analysis of tumour burden. This plot illustrates the coefficients for the interaction terms with treatment weeks; thus, these represent factors that change the slope of the disease burden curve. The units for these coefficients are log10 n-fold change of radiant flux per week. The baseline condition represents mice with an initial flux of 10⁶ p/s (photons/second) in the control group, although the baseline for a given animal is assumed to vary (random intercepts model). The full model specification can be found in Table A2. (**b**) Logistic regression model predicting probability of progressive disease. Progressive disease was defined by a statistically and clinically significant increase in the disease burden (see definition in the methods in the Appendix A). The odds ratio represents the change in the odds of progressive disease when the relevant variable is true (compared to the baseline case). The intercept for this model represents the baseline odds of progressive disease. See Table A3 for full model specification. (**c**) This subgroup analysis focusses on mice with recurrent disease. Refer to Table A4 for full model specification.

The trajectories of the tumour burden of each individual over time may be summarised using a spaghetti plot. Using the mixed-effects model for longitudinal analysis of tumour burden, we found that higher residual tumour burden post-resection significantly increased the rate of progression, while both G and HiCi treatments reduced the rate of progression of disease (Figure 1a; Table A2). However, G and HiCi treatments appeared to be non-additive. 

As the above analysis may be influenced by the relative proportions of mice that were “cured” by surgical resection (i.e., no residual disease at the commencement of adjuvant treatment), treatment effects were analysed by two further methods. First, a logistic regression model of the probability of progressive disease (defined in the Appendix A) was constructed. This demonstrated that the odds of developing progressive disease was significantly reduced with HiCi treatment (OR 0.15 (95% CI 0.0250 to 0.86). *p* = 0.033) but not with G treatment (OR 0.80 (95%CI 0.172 to 3.68). *p* = 0.771) (Figure 1b; Table A3).

Second, longitudinal analysis was performed on the subset of animals that harboured residual disease and thus would potentially respond to adjuvant therapy. Statistical modelling of this subset of mice confirmed treatment effects of HiCi but not G treatment (Figure 1c; Table A4). The rates of progression of disease as predicted by this multivariate model are shown in Table A4b. When reported as mean tumour burden doubling times (as measured by radiant flux), the HiCi group had the longest doubling time (18.5 days), indicating substantially slowed progression compared to the Ctrl (10.6 days), G (14.1 days) and G+HiCi (13.6 days) groups. The spaghetti plot of this subgroup analysis is shown in Figure A6.

HiCi treatment did not significantly influence body weight changes, while G treatment led to progressive mild weight loss, possibly suggesting G, but not HiCi, exhibited treatment toxicity. There were no other physiological or behavioural side effects observed, beyond those associated with progressive cancer.

#### 3.1.2. HGF/c-Met Inhibition Is Associated with Reduced Tumour Vascularity

**Figure 2:**
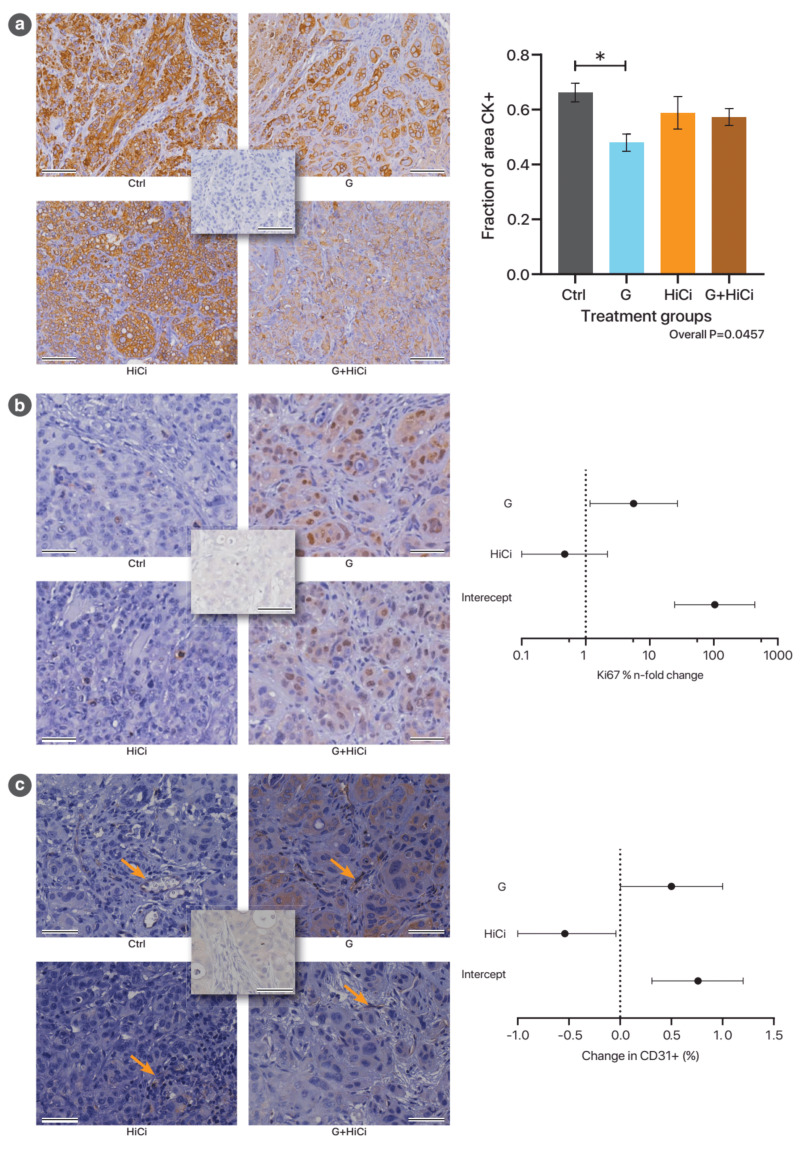
Summary of immunohistochemistry of local recurrent disease. (**a**) Left: Representative images for pan-cytokeratin staining for tissue sections of the recurrent tumour from the different treatment groups. The central image represents the isotype negative control. Images were acquired with 20× objective. Scale bars: 100 µm. Right: Column graph representing the relative cancer cell density (pan-cytokeratin staining) of the four treatment groups. * *p* < 0.05. (**b**) Left: Representative images for Ki67 staining for the different treatment groups. Note that it is the nuclear staining that is important (cytoplasmic background signals are noted in G and G+HiCi groups). The central image represents the isotype negative control. Images were acquired with 20× objective. Scale bars: 50 µm. Right: Graphical representation of the linear regression model of Ki67 positivity versus treatments. Model specification may be found in Table A5. (**c**) Left: Representative images for endothelial (CD31) staining of recurrent tumours from the different treatment groups. Selected endothelial cells staining for CD31 are indicated with arrows. The central image represents isotype negative control showing no endothelial staining over an area which appears to contain capillaries. Images acquired with 40× objective. Scale bars: 50 µm. Right: Graphical representation of the linear regression model of CD31 staining (as a percentage of tumour cross-sectional area) versus treatments. Model specification may be found in Table A6.

Immunohistochemistry performed on local recurrent disease demonstrated that the cytokeratin positive cancer cell fraction was decreased in the gemcitabine treated tumours compared to controls (*p* = 0.032), while Ki67 positive cells per cancer cell area was significantly increased by gemcitabine treatment, suggesting G treatment may select out a subset of cells with higher proliferative potential (Figure 2a,b). Gemcitabine treatment was also found to increase vascularity of tumours (increased CD31 per unit area) compared to controls, while HiCi treatment was associated with reduced vascularity, suggesting an anti-angiogenic effect (Figure 2c). Neither apoptosis (TUNEL staining) nor stemness (DCLK1 staining) demonstrated statistically significant differences between treatment groups.

#### 3.1.3. Circulating Tumour Cells as a Marker of Recurrence

**Figure 3:**
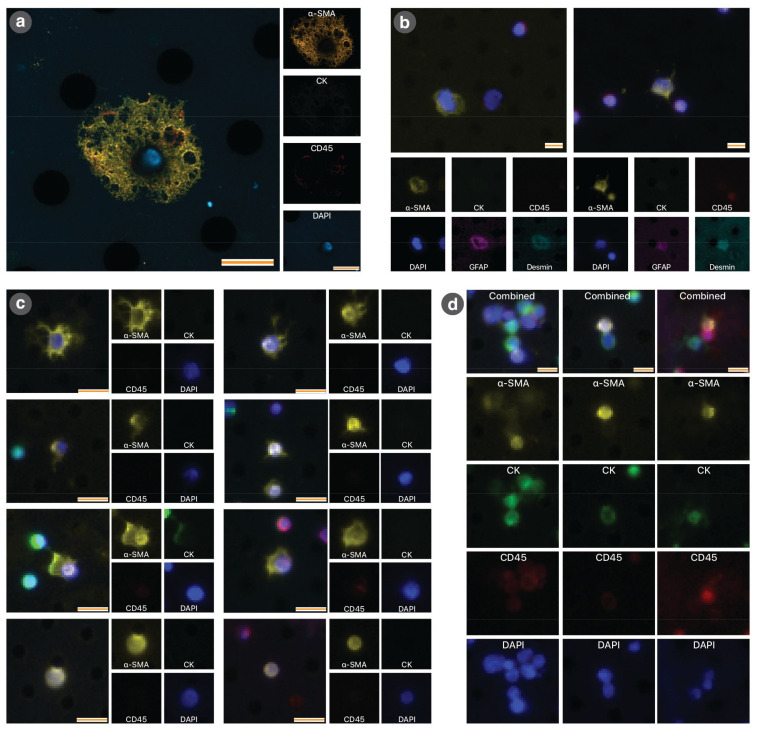
Immunocytochemical characteristics of circulating pancreatic stellate cells. (**a**) High-powered confocal microscopy image of cPSCs. The cPSC depicted demonstrates ovoid morphology and positive staining for α-SMA and DAPI but negative staining for CK and CD45. Small images on right represent individual fluorescence imaging channels. (**b**) Representative images of cPSCs that have undergone quenching and re-staining, demonstrating CK−/CD45−/α-SMA+/Desmin+/GFAP+/DAPI+. The larger image at the top is a combined four-channel image (α-SMA/CK/CD45/DAPI). The smaller images at the bottom are pseudo-colour images of each channel, including images acquired after quenching and re-staining (GFAP and desmin). The left cPSC demonstrates an ovoid morphology whereas the right cPSC demonstrates a more spiculated morphology. Note the size difference between adjacent CD45+ staining leucocytes. (**c**) Representative images of the four major morphologies of cPSCs identified on wide field microscopy. Images taken using 10× objective. There are two images for each morphology (arranged side to side). The smaller images represent the corresponding individual fluorescence imaging channels. The four morphologies were: (i–ii) Stellate morphology (St): well defined cytoplasmic extensions creating a spiculated appearance. (iii–iv) Round morphology with cytoplasmic extensions (CE): small round cells, with impressions of cytoplasmic extensions. (v–vi) Ovoid (Ov): large cells with prominent cytoplasm without definite spicules. (vii–viii) Round and bland morphology (RB): round cytoplasm without any evidence of extensions. (**d**) Three examples of heterotypic clusters containing cPSCs. Scale bars for all images: 10 µm.

A mean ± SE of 340 ± 6.7 μL of portal vein blood were collected from 54 animals for analysis of circulating rare cells. CTCs were identified as CK+, CD45− and aSMA− cells. Circulating PSCs were identified as CK−, CD45− and aSMA+ cells (Figure 3a). As noted under Methods, the identities of PSCs were confirmed by positive expression of the PSC-selective markers desmin and GFAP (Figure 3b). Interestingly, we observed that cPSCs could be subcategorised morphologically (by wide-field microscopy) into four groups: stellate (St), rounded with cytoplasmic extensions (CE), ovoid (Ov) and rounded and bland (RB) (Figure 3c). These morphological types differed in size and circularity, but not staining intensity for α-SMA (Figure A7). We also noted that while most cPSCs existed as individual cells, a number of cPSCs were found in heterotypic cell clusters containing both cPSCs and CTCs (Figure 3d). The significance of such clusters was not investigated.

**Figure 4:**
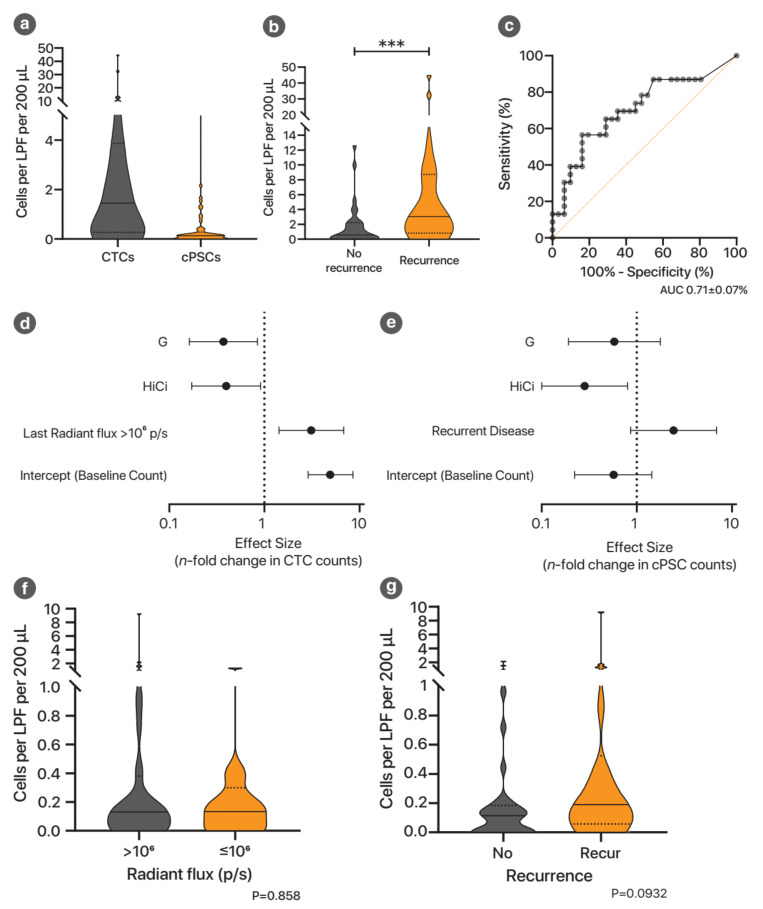
Effect of tumour burden and treatments on circulating tumour cells and circulating pancreatic stellate cells. (**a**) Violin plot of circulating rare cell numbers. This includes all mice, regardless of treatment group allocation. Solid transverse line represents the median. Dotted lines represent the first and third quartiles. **(b**) CTC counts in animals with and without recurrence, as defined by the combination criteria. *** *p* < 0.001. (**c**) Receiver operator characteristic curve describing the performance of CTC counts as a test to determine recurrent disease. Different points in the curve represent different cut-offs for CTC counts. The area under the curve (AUC) represents the effectiveness of the test in discriminating recurrence. AUC values range from 0.5 (not better than chance) to 1.0 (perfect discriminatory test). (**d**) Poisson regression model of CTC counts versus treatments. This model assesses the effects of treatments on CTC counts, taking into account the last measured tumour burden (assessed by bioluminescence imaging). The full model specification is shown in Table A7. (**e**) Poisson regression model of portal vein cPSC counts versus treatments. This model assesses the effects of treatments on cPSC counts, taking into account the tumour burden at the end of the experiment (as measured by bioluminescence flux). The model specification is shown in Table A8. (**f**) Violin plots comparing cPSC counts in animals with high tumour burden versus low tumour burden at the end of the experimental period (as determined by bioluminescence imaging). This suggests that the observed higher cPSC counts in mice with recurrent disease is not due simply to higher disease burden at the time of blood sampling. (**g**) Violin plots comparing the portal vein cPSC counts for animals with and without recurrence. This demonstrates a trend for higher cPSC counts to be associated with mice with recurrent disease post-resection.

All circulating rare cell (CTC/cPSC) counts reported herein represent the count per low-powered field (LPF) per 200 μL of portal vein blood. The median (IQR) number of CTCs detected was 1.5 (0.28 to 3.8) but ranged up to 45 (Figure 4a). Nine (17%) of the specimens did not yield any CTCs. 

Animals with histological or bioluminescence evidence of recurrence were found to have higher CTC counts compared to animals without (median count 3.1 vs. 0.57; *p* = 0.0099) (Figure 4b). Receiver operator characteristic (ROC) curve analysis demonstrated an area under the curve (AUC) of 0.71 (95% CI 0.56 to 0.85), suggesting that CTC count was a fairly good test to determine the presence of tumour recurrence (Figure 4c).

#### 3.1.4. Both G and HiCi Treatments Reduced CTC Counts

The median CTC counts were 4.2, 0.81, 2.0 and 0.76 for control, G, HiCi and G+HiCi groups, respectively, suggesting a possible treatment effect by both gemcitabine and AR. Poisson regression demonstrated that, after taking into account tumour burden, both G and HiCi treatment were indeed associated with lower numbers of CTCs (Figure 4d). High tumour burden (as represented by the last measured ventral radiant flux prior to necropsy), not surprisingly, was also associated with a higher CTC count. 

#### 3.1.5. HiCi Treatment Reduced Circulating Pancreatic Stellate Cells (cPSCs)

Putative cPSCs were identified using immunocytochemistry: α-SMA+ (α-smooth muscle actin, PSC activation marker), CK− (pan-cytokeratin, epithelial/cancer marker), CD45− (leucocyte marker) and DAPI (nuclear staining) (Figure 3a and Figure A4). The median (IQR) number of cPSCs detected was 0.13 (0 to 0.22) per LPF per 200 μL of portal vein blood but ranged up to 9.3 (Figure 4a). Seventeen (32%) of the specimens did not yield any cPSCs. Statistical modelling found that HiCi treatment was associated with reduced cPSC counts, whereas gemcitabine treatment had no significant effect (Figure 4e). Unlike CTC numbers, the cPSC count was not associated with the tumour burden, as measured by the last radiant flux measurement (Figure 4f). Tumour recurrence demonstrated a non-significant trend for increasing the cPSC count (Figure 4g). 

### 3.2. Transcriptomic Characterisation of cPSCs

To further characterise cPSCs, single-cell transcriptomic analysis was performed (Model 2). Two mice with orthotopically implanted pancreatic cancer were allowed to progress for 8 weeks. The mice were euthanised and samples of the primary tumour (MsPrim), metastasis (MsSec) and blood (MsBlood) were collected and pooled. Cultured cancer-associated human PSCs and AsPC-1 cells (Cult)—which were the source of implanted cells—were used for comparison. Single-cell RNA sequencing was performed; the basic sequencing and alignment metrics are shown in Table A1. 

#### 3.2.1. Circulating PSC Identities Confirmed by ScRNA-seq

**Figure 5:**
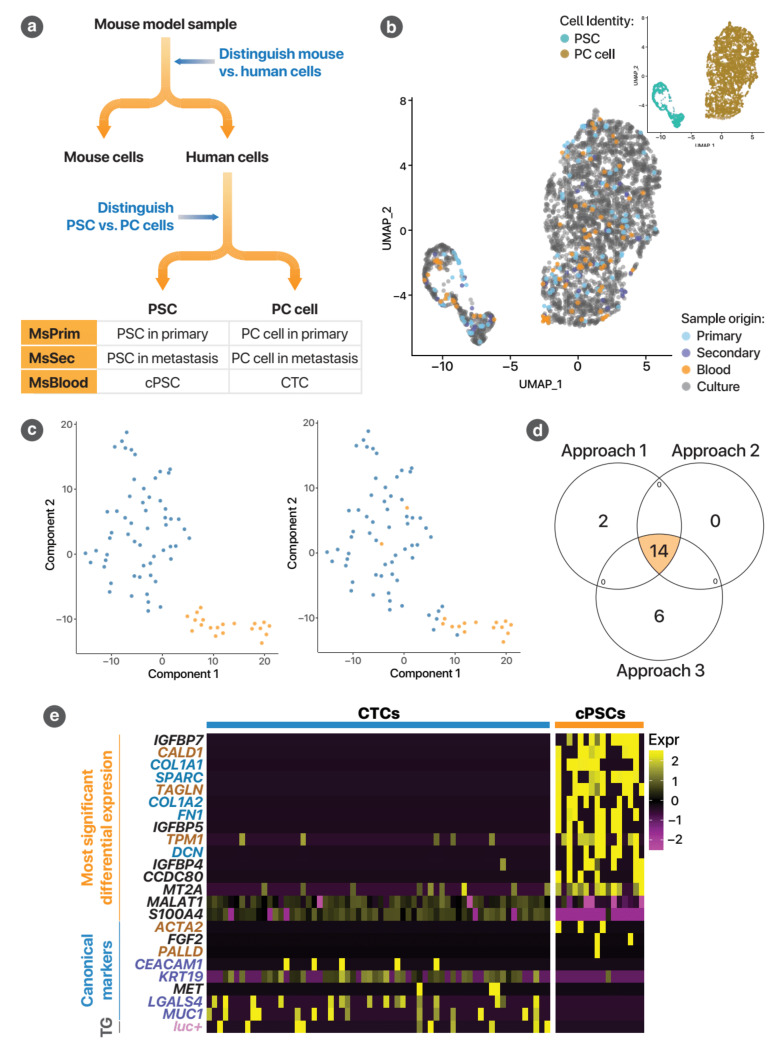
Identification of circulating pancreatic stellate cells using single-cell RNA-sequencing. (**a**) General circulating PSC identification strategy. The only human cell types implanted into the mice were AsPC-1 cancer cells and PSCs. Thus, by identification of human cells, one can narrow down the identity of cells to these two cell types. Circulating rare cells may then be identified by combining the cell type identity with the sample of origin (for instance, human cells in MsBlood must be circulating tumour cells (CTCs) or circulating PSCs). (**b**) Integration of human cells across the four samples for Approaches 1 and 2. This demonstrates that human cells from all four samples clustered into two distinct groups, representing PC cells and PSCs. Inset: The same plot, colour coded for presumptive cell type based on the UMAP co-ordinates of the cells (Approach 1). (**c**) Approach 3 avoids the assumptions of data integration. Instead, using cultured AsPC-1 and PSCs as reference, the top 500 genes predictive of the identity of each cell type was identified (i.e., 1000 genes in total). This was applied to the human cells within the blood sample to classify the cells using unsupervised clustering. Left panel: t-SNE plot showing the two clusters of cells representing CTCs and circulating PSCs. This approach yielded 20 circulating PSCs. Right panel: The same plot, coloured by the classification results from Approach 1, indicating good agreement between the two approaches. (**d**) This is a Venn diagram summarises the degree of agreement between the three approaches described. The overall agreement between each approach was high. There were 14 cells which were identified by all three approaches, which represent the cells identified with high confidence as circulating PSCs in this experiment. (**e**) This heatmap demonstrates the differences in gene expression between CTCs and circulating PSCs identified using Approach 1. The upper half of the heatmap (marked on the left with orange) represents the most significant differentially expressed genes (false discovery rate < 10%). This is followed by other known canonical markers (blue) and finally the luciferase transgene. The gene names are also colour-coded. Brown represents genes for myofibroblast or cytoskeletal proteins associated with PSCs. Blue represents extracellular matrix proteins associated with PSCs. Purple represents epithelial proteins associated with PC cells. Pink represents the luciferase transgene of AsPC-1 cells used in this experiment.

To identify circulating PSCs, we first distinguished human cells from mouse cells. The human cells thus identified could only have two identities, PSCs or PC cells (Figure 5a). The first step utilised relative unique molecular identifier (UMI) counts (Figure A8a). This was performed using relative UMI counts of mouse to human genes, with accuracy of this confirmed by cell classification based on gene expression of orthologous genes (Figure A8b) [20]. Mouse and human cells were further classified using canonical markers (Figure A9).

**Figure A5:**
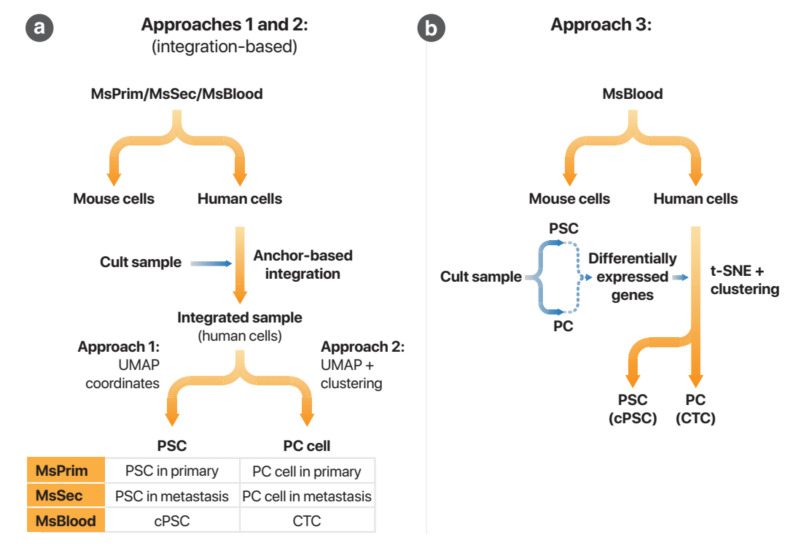
Different approaches to cPSC identification. These flow charts summarise the three different approaches used for cPSC identification. (**a**) Approaches 1 and 2 relies on an anchor-based integration technique to combine the human cells of the three mouse model samples with the human cultured cell samples. Cell identification was then performed either according to UMAP coordinates (Approach 1) or by unsupervised clustering (Approach 2). (**b**) Approach 3 avoids integration of samples altogether. First, the cultured cell sample was used to derive a list of 1000 differentially expressed genes between PC cells and PSCs (top 500 markers for each cell type). These genes were then used to classify the MsBlood human cells into either PC cells (CTCs) or PSCs (cPSCs) by unsupervised clustering.

**Figure A6:**
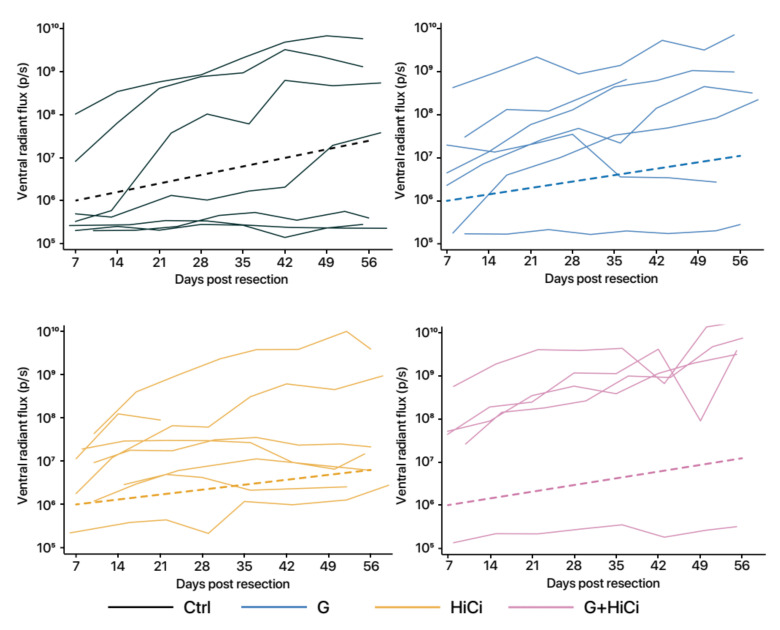
Spaghetti plot demonstrating the trajectories of tumour burden over time in the subgroup of mice with recurrent disease. The solid lines represent the actual trajectories, while the dotted line represents the predicted trajectory for a mouse with an low residual disease post resection (initial flux of 10^6^ p/s) for a given treatment group. This predicted trajectory is based on the statistical model tabulated in Table A4 and illustrated in Figure 1c. Note that the ventral radiant flux, a surrogate measure of total disease burden, is in logarithmic scale. Therefore, a linear trajectory represents exponential tumour growth, and each unit represents a 10× increase in radiant flux, thus a small change in the gradient of the curve represents a significant change in the progression of disease of a mouse. Note also that the gradient of the trajectories increases as the initial radiant flux increases, which is reflected in statistical model. While high levels of residual disease post resection results in rapid progression of disease in all groups, in the HiCi group, mild to moderate disease burden resulted in a relatively flat trajectory, reflecting the significant treatment effect found in the statistical model.

**Figure A7:**
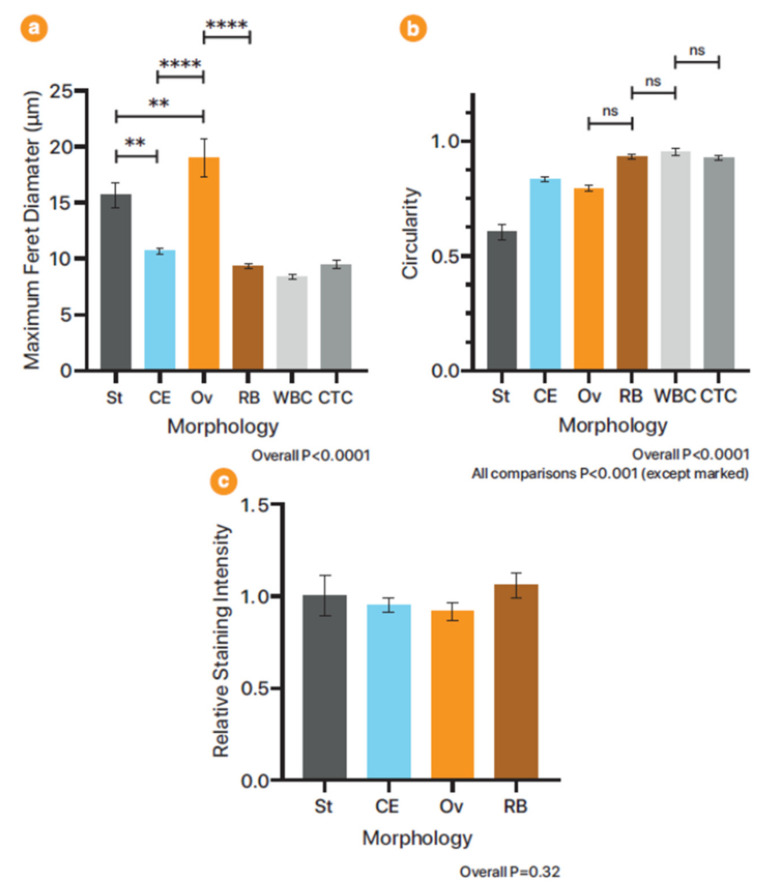
Selected measurements for cPSCs according to morphology type. (**a**) Maximum Feret diameter. This is the caliper diameter of the largest dimension of the cell. There was an overall significant difference (*p* < 0.0001 on ANOVA). The significant differences between groups (by Tukey’s multiple comparison test) are highlighted by the capped lines. Leucocyte (WBC) and CTC diameters are shown for comparison. For clarity, *p*-Value for comparisons with these cells are not shown. (**b**) Circularity. This is calculated by the formula circularity = 4 π × (area / perimeter^2^). A value of 1.0 represents a perfect circle. As the value tends towards zero, it represents an increasingly elongated polygon. All multiple comparisons were statistically significant (*p* < 0.0001) except for the comparisons indicated. (**c**) Relative staining intensity for α-SMA. This represents the staining intensity for each cell in the α-SMA channel relative to the mean of all measurements. There were no significant differences between the groups. Abbreviations: WBC, white blood cell. Abbreviations for morphologies described in the text. ** *p* < 0.01, **** *p* < 0.0001.

**Figure A8:**
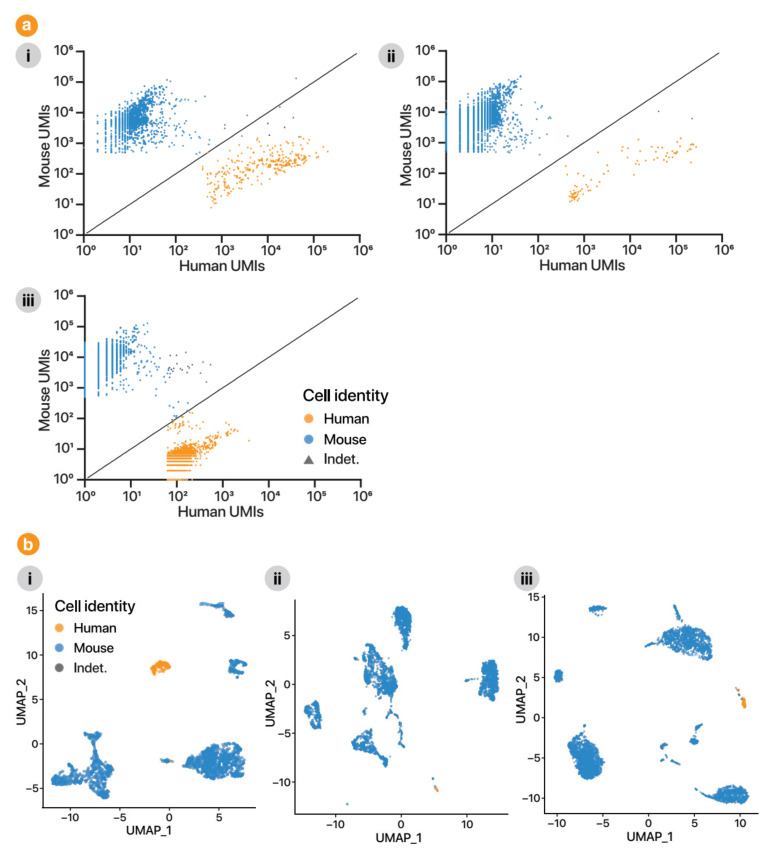
Classification of mouse versus human cells. (**a**) Classification by relative UMI counts. These plots the number of mouse UMIs to human UMIs for each cell (barcode) in the mouse model samples: (**i**) MsPrim, (**ii**) MsSec, and (**iii**) MsBlood. This demonstrates that there is little overlap of the two groups of cells when considering the proportion of UMIs mapped to mouse and human genomes for classification of host species cells. Note: Cells with UMIs mapped exclusively to one or the other reference genomes could not be plotted in log scale, and are therefore omitted from these plots. (**b**) Classification by gene expression. Using only one-to-one orthologous genes to classify the cells, the UMAP plot clearly indicates human cells clustering away from mouse cells. The colour represents the identity of the cell as determined by relative UMI counts (as in [a] above). These UMAP plots demonstrate a high degree of agreement between the two classification strategies, thus validating the cell type identification. (**i**) UMAP plot for MsPrim sample. (**ii**) UMAP plot for MsSec sample. (**iii**) UMAP plot for MsBlood sample. Abbreviations: Indet., indeterminate.

**Figure A9:**
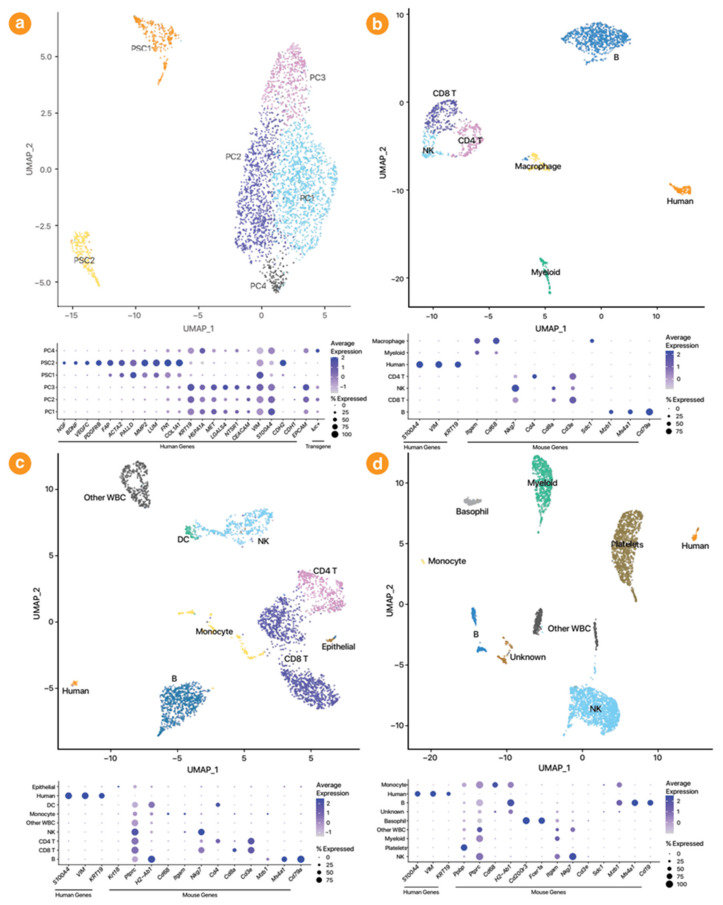
Cell Clustering and Identification. (**a**) UMAP and dot plots of cells from the mouse blood sample (MsBlood), demonstrating clustering into two major cell types (PSCs and PC cells) and illustrating canonical marker expression of these cell subgroups. Luciferase (*luc+*) was expressed in some PC cells but not in PSCs, as expected. (**b**–**d**) UMAP and dot plots, demonstrating clustering into different cell types and relative canonical marker expression. Cells which expressed the leucocyte marker *Ptprc* (CD45) without classical leucocyte subtype expression profile were classified as “Other WBC”. The specimens are: (**b**) mouse primary tumour sample (MsPrim); (**c**) mouse model metastatic tumour sample (MsSec); (**d**) mouse model blood sample (MsBlood). Abbreviations: B, B lymphocytes. DC, dendritic cells. NK, natural killer cells. T, T lymphocytes (CD4+ or CD8+). WBC, white blood cells.

**Figure A10:**
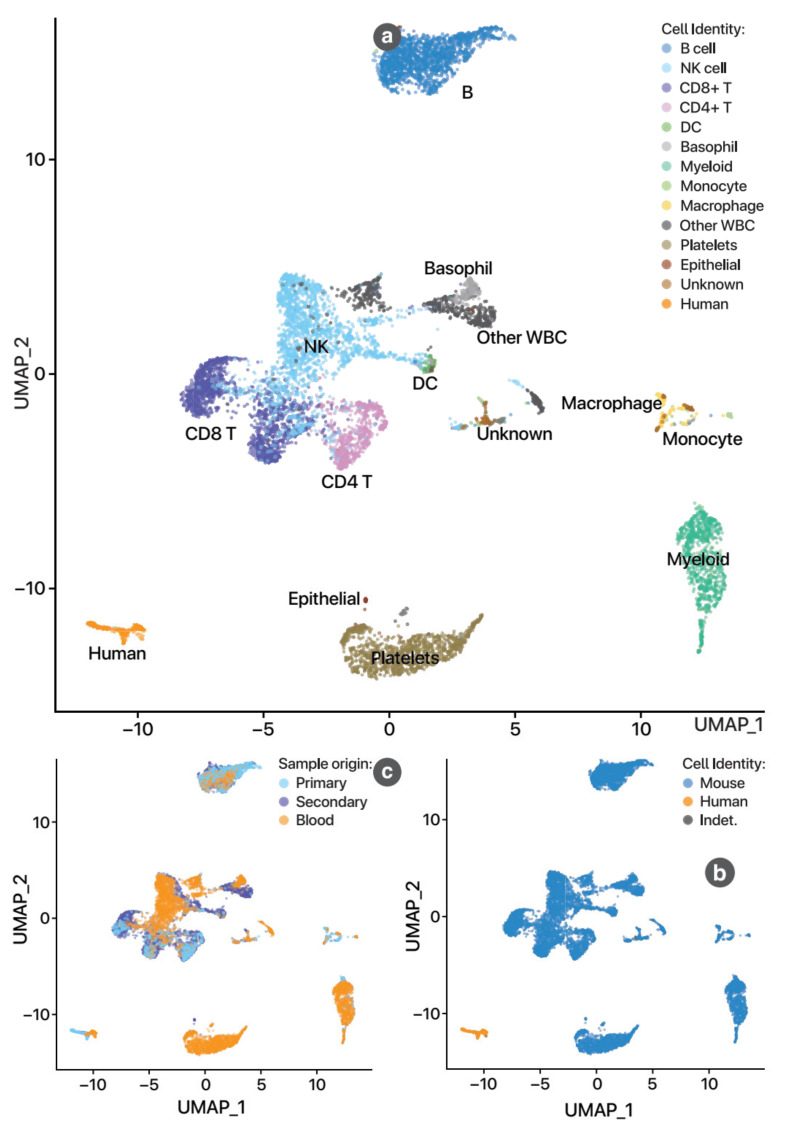
Successful integration of gene expression data across samples. These set of figures demonstrate cells clustered according to gene expression (i.e., cell type) (**a**), rather than to sample origin (**b**). Human cells clustered separate from mouse cells, as expected (**c**). Abbreviations: B, B lymphocyte. DC, dendritic cell. Indet., indeterminate. NK, natural killer cell. T, T lymphocyte. WBC, white blood cell.

The number of human cells available for analysis in the four samples, after filtration for quality, were 100 (MsPrim), 40 (MsSec), 75 (MsBlood) and 2791 (Cult). From these cells, cPSCs and CTCs were identified using three different bioinformatics approaches (Figure A5). In the first two approaches, human cells from each of the four samples were integrated using an anchor-based method. Successful integration of gene expression data was achieved (Figure A10). The resulting UMAP (uniform manifold approximation and projection) plot indicated that these human cells clustered with the two cultured cell types (PSCs and PC cells) (Figure 5b). Those PSCs originating from the MsBlood sample were classified as cPSCs. Sixteen cPSCs were identified based on UMAP coordinates (Approach 1) and 14 cPSCs were identified using unsupervised clustering (Approach 2). A third approach, avoiding data integration altogether, was used to confirm these findings (Figure A5b). This utilised the differential gene expression between PSCs and AsPC-1 cultured cells (i.e., parental cells) to identify cells in mouse samples. This yielded 20 cPSCs and 58 CTCs (Figure 5c). 

**Figure A11:**
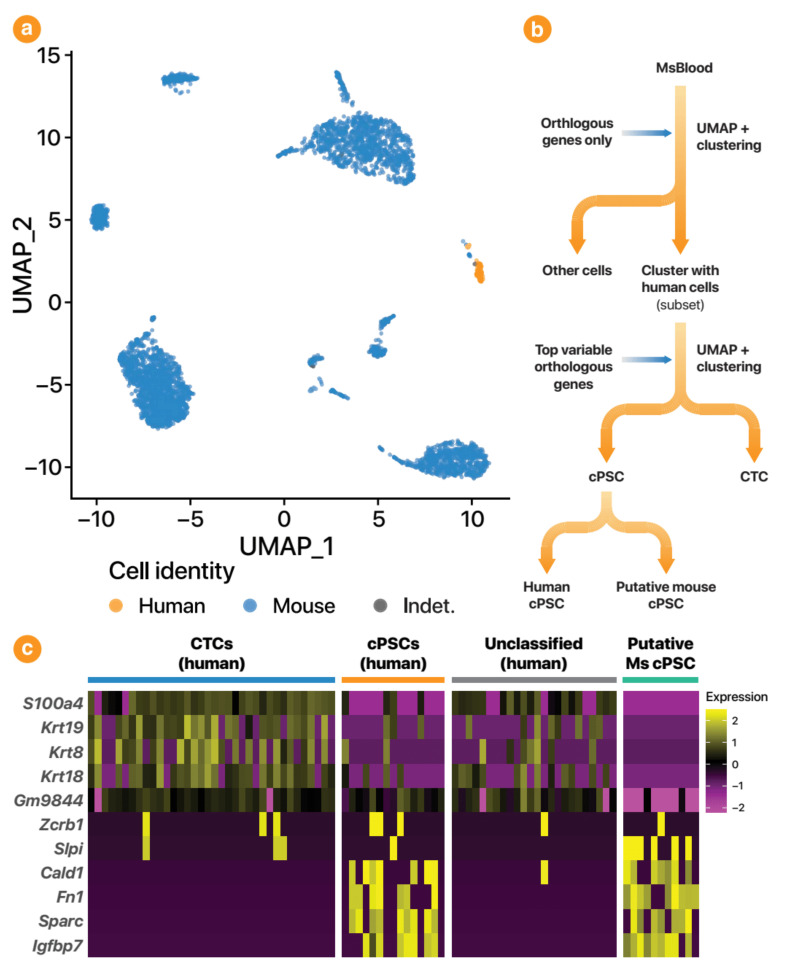
Potential mouse cPSCs in MsBlood sample. (**a**) Gene expression analysis restricted to one-to-one orthologous genes of cells in the MsBlood sample is summarised in the UMAP plot above. Clustering close to the human cells are a number of mouse cells which possibly represent mouse cPSCs. (**b**) This subset of cells were analysed by unsupervised clustering into three groups: PSCs, CTCs and unclassified (non-specific expression pattern). (**c**) Combining the results of cell type classifica-tion with the classification of cell species (mouse versus human), one can infer the identities of putative mouse cPSCs. Abbreviations: Ms, mouse. Indet., indeterminate.

The high degree of agreement between the three independent approaches (Figure 5d) strongly supports the existence of cPSCs in our orthotopic mouse model of PC. The identification of putative mouse cPSCs provides further supportive evidence for their existence (Figure A11).

The pattern of differential gene expression between these two cell types supports their presumptive identities. Figure 5e shows the gene expression of the 62 CTCs and 16 cPSCs classified using the first approach. Circulating PSCs expressed higher levels of genes associated with extra-cellular matrix proteins (gene names in blue in the figure: *COL1A1*, *SPARC*, *COL1A2*, *FN1*, *DCN*) and myofibroblast cytoskeletal proteins (in brown in the figure: *CALD1*, *TAGLN*, *TPM1*, *ACTA2*, *PALLD*), whereas CTCs expressed epithelial and cancer markers (in purple in figure: *CEACAM1*, *KRT19*, *LGALS4*, *MUC1*). Importantly, the transgene for luciferase, only present in the implanted luciferase-tagged AsPC-1 cancer cells (*luc+*, in pink in the figure), was not expressed by any of the putative cPSCs, whereas a proportion of CTCs did express it at detectable levels. This non-detection of the luciferase transgene expression was statistically significantly different from the luciferase expression of all cancer cells across the samples (*p* = 0.0030). This strengthens the conclusion that these putative cPSCs are not cancer cells.

#### 3.2.2. Potential Pathways Influencing cPSCs

**Figure 6:**
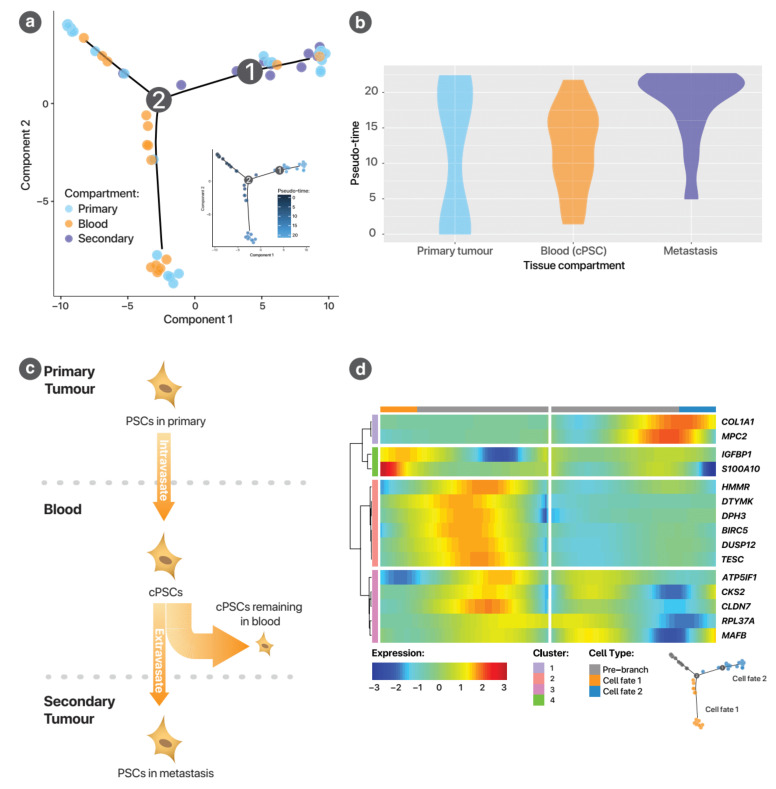
Trajectory analysis of PSC development. (**a**) This shows the PSC development trajectory, with cells coloured to reflect the sample of origin, demonstrated a bifurcated trajectory, with one major branch point (vertex 2). The inset figure shows the same trajectory, with cells shaded by pseudo-time. At the origin of the trajectory, PSCs from the primary cancer predominate. The two limbs arising from vertex 2 lead to different cell fates: one dominated by circulating PSCs (lower limb) and one by PSCs in the metastasis (right limb). (**b**) Pseudo-temporal changes in cell numbers by compartment. These violin plots present the changes in the numbers of cells in each of the tissue compartments over pseudo-time. The most important observation here is the relative timing of the peaks of cell numbers in the different compartments: first primary cancer, then blood and finally metastasis. This is consistent with the biology of the metastatic process. Note that the width of the violin plots only reflects relative cell numbers within the compartment, not between compartments. The scaling of the violin is such that the total area under the curve is the same for each violin (i.e., it is a probability density function for that sample). (**c**) This panel shows an interpretation of the PSC trajectory. PSCs intravasate into the blood to form cPSCs. A small proportion of these circulating PSCs extravasate and incorporate into metastatic nodules (or form metastatic niches). The remainder remain in the circulation. This explains the bifurcated trajectory. (**d**) This heatmap describes changes in gene expression over pseudo-time as cells pass through branch point 2. The centre of the heatmap represents the pre-branch trajectory, which is signified by the grey bar above and the grey cells in the legend trajectory map below. The two sides of the heatmap represent the two cell fates (Cell fate 1, predominated by cPSCs, in orange; and Cell fate 2, predominated by metastatic PSCs, in blue). Genes are clustered by *k*-means clustering.

**Figure A12:**
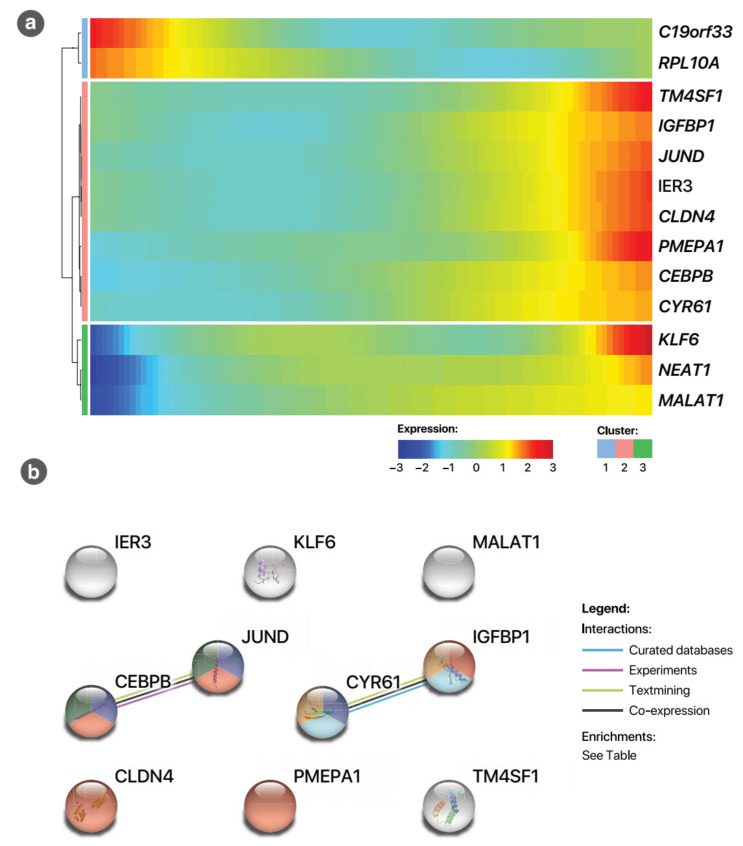
Changes in gene expression with pseudo-time. (**a**) This heat map summarises the genes in PSCs whose expression varied significantly over pseudo-time. (**b**) The same genes represented as a protein network. Each circle (“node”) represents a protein product from the gene while the connecting lines (“edges”) represent interactions between these proteins. The nodes summarises the genes/proteins involved in the enriched pathways listed in Table A9. The colour of the edges represents the nature of the evidence of these interactions (as reported by the STRING database). Overall interaction enrichment *p*-value was 0.0353, suggesting this group of over-expressed genes are likely to be biologically connected.

To understand the role of PSCs and to identify potential pathways for future study, trajectory analysis was performed on PSCs across the mouse model samples (MsPrim, MsSec, MsBlood) using the Monocle package, as described further in the Appendix A. Trajectory analysis orders cells according to their relative gene expression by assuming the cells represent different states along a development pathway (*trajectory*). The inferred passage of time over this pathway is called *pseudo-time*. Figure 6a shows that the trajectory takes on a biologically plausible progression from a state dominated by primary cancer PSCs (upper left), then cPSCs (lower limb) and finally, metastasis (upper right). This is supported by the progressive peaks of cells from the primary tumour, blood and secondary tumour over pseudo-time (Figure 6b). A possible interpretation of this trajectory is shown in Figure 6c. Genes with significant changes in their expression over pseudo-time (FDR < 0.05) of this trajectory is shown in Figure A12. The gene ontology process, response to endogenous stimulus, was found to be enriched (FDR = 0.0345). This included genes *IGFBP1*, *CLDN4* (involved in tight junction interactions) and *PMEPA1* (involved in transforming growth factor β (TGF-β) receptor signalling pathways) (Table A9).

**Figure A13:**
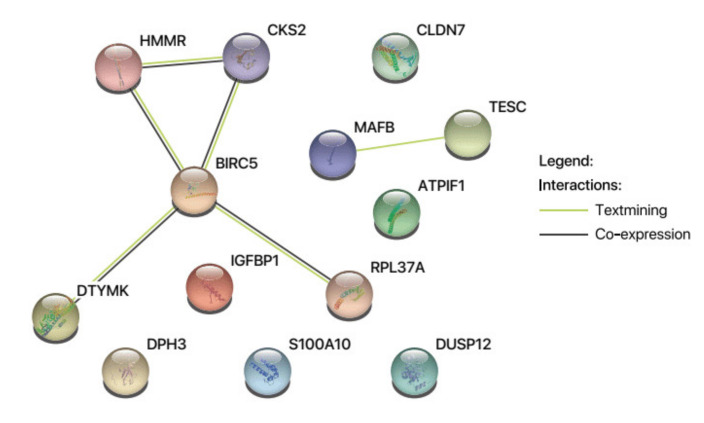
Genes upregulated in cPSCs as protein network. Each node represents a protein product from the gene while the edges represent interactions between these proteins. Overall interaction enrichment *p*-Value was 0.00144, suggesting this group of over-expressed genes are likely to be biologically connected.

To explore the changes in PSCs depending on their cell fate at the major branch point where some cells become metastatic cells (i.e., localised within metastases) and some remain in the blood as circulating PSC, branch point analysis was performed. Figure 6d summarises the genes that were differentially expressed (FDR < 0.05) between cells passing down the two cell fates (cPSCs and metastatic PSCs). The centre of the heatmap represents the pre-branch state, while the left and right margins represent the two different cell fates. Changes in gene expression is represented by the heatmap and passage of pseudo-time represented by the distance from the centre. The two genes that were upregulated as cells passed into the metastatic cell fate were *COL1A1* and *MPC2*, whereas 13 genes were upregulated as cells passed down the trajectory towards cPSCs (Figure 4c). STRING network analysis of these 13 upregulated genes related to cPSC cell fate demonstrates six interactions (*p*-Value = 0.00144) (Figure A13).

## 4. Discussion

This study is the first to demonstrate that the use of HGF/c-Met in the adjuvant setting resulted in the inhibition of PC progression post-resection. This paper also provides the first confirmation of the presence of circulating PSCs and describes their modulation by adjuvant therapy for PC. 

### 4.1. HGF/c-Met Inhibition as Adjuvant Treatment

The major finding of this study was that concomitant inhibition of both the ligand and receptor of the HGF/c-Met pathway in an adjuvant setting significantly reduced the rate of progression of recurrence and allowed the mice to maintain a stable disease burden over the observation period. This occurred even in mice with a moderate degree of residual disease post-resection.

We previously demonstrated that HGF/c-Met pathway inhibition is effective at minimising and even eliminating metastasis in early as well as advanced models of pancreatic cancer [8,9,10]. Our novel data raise the interesting possibility that the mechanisms for this prevention of metastasis with HGF/c-MET inhibition involve reduction of circulating PSCs. 

The therapeutic use of HGF/c-Met pathway inhibitors is not new. A number of phase III trials of selective HGF or c-Met inhibition have failed to demonstrate drug efficacy [21,22,23]. The development of rilotumumab, the HGF inhibitor used in this paper, has largely been abandoned since the unsuccessful RILOMET-1 trial [24]. The trial was terminated after the rate of fatal adverse events due to disease progression was observed to be higher in patients randomised to rilotumumab (in combination with ECX (epirubicin, cisplatin, capecitabine) chemotherapy) group. Importantly, however, the frequency of serious adverse events as well as fatal adverse events, not due to disease progression, was not significantly different between the treatment groups [24]. 

However, there are a number of reasons why these negative human trials should not preclude the use of the proposed two-pronged HGF/c-Met inhibition approach for the treatment of human PC. (i) It must be recognised that *none* of these previous clinical trials were performed on patients with PC. PC is well known to overexpress HGF as well as c-Met, both in patients and in cell lines [25,26,27], and this overexpression expression is known to be associated with poorer survival [28]. (ii) The treatment approach used in these previous trials differed from the treatment employed in this model. The RILOMET-1 trial inhibited the HGF/c-Met pathway by rilotumumab only. Rilotumumab alone has been shown, in our group’s work, to be less effective than combination HGF and c-Met inhibition [9,10]. (iii) The clinical trials in non-small cell lung cancer have utilised HGF *or* c-Met inhibition exclusively to inhibit this pathway (generally in combination with an epidermal growth factor receptor inhibitor). Dual inhibition of both the ligand and receptor of the HGF/c-Met pathway has the theoretical advantage of overcoming incomplete neutralisation of HGF. This has not yet been the subject of clinical trials in humans and there is no evidence of emerging clinical trials using this approach—no trials utilising a two-pronged approach to HGF/c-Met inhibition are currently registered with ClinicalTrials.gov. 

Taken together, the findings of the current study and our previous publications strongly suggest efficacy for HGF/c-MET inhibition ± chemotherapy in early, advanced and adjuvant preclinical settings, thus presenting a strong case for clinical trials of this treatment strategy in patients with PC.

### 4.2. Circulating PSCs—A Novel cellular Intermediary for PC Metastasis?

The second major finding of this paper is the discovery of cPSCs. This has the potential of providing a new perspective in our understanding of the metastatic progression of pancreatic cancer. PSCs are known to play an important role in the development of PC [29]. While there have been some reports suggesting a “protective” effect of PSCs, the weight of available evidence points to a facilitatory function for these cells in cancer progression [30,31,32]. The pro-metastatic effect of PSCs, as demonstrated in co-injection mouse models, suggests a role beyond just local primary tumour effects [14,33]. It is also in these co-injection models, using human cancer cells and human PSCs, that the first evidence of possible PSC migration was found, as evidenced by co-localisation of human nuclear antigen and α-SMA in metastatic deposits [14]. Subsequently, the definitive evidence for the capacity of PSCs to migrate was reported by our group through the use of a gender mismatch study [12]. 

The existence of these PSCs at distant sites must imply the existence of a route of dissemination. Possible routes include the classical pathways of spread of malignancy (haematogenous, lymphatic, transcoelomic). The disparate sites of metastasis, especially beyond the peritoneal cavity (for instance, lung), suggest the former two, thus first hinting at the existence of cPSCs.

In this study, circulating PSCs were identified using a range of well-established PSC-selective markers and by confirming that these cells did not exhibit cancer cell or leucocyte markers. Strong evidence that our findings are not “chance” observations is provided by the following observations: (i) cPSCs could be reliably identified in two separate mouse models of PC; (ii) cPSCs were identified using both immunohistochemistry and single-cell RNA sequencing; (iii) cPSC numbers were found to vary in a way that is consistent with their postulated pathophysiology; (iv) cPSCs were modulated by drugs which strongly inhibit metastatic disease (HGF/c-Met inhibition [8,9,10]); and (v) cPSCs tend to be more commonly found in mice with recurrent disease.

An alternative explanation of the observed cPSCs is the presence of CTCs which have undergone EMT (epithelial to mesenchymal transition). Such EMT CTCs, reported in a variety of cancers, are characterised by the loss of epithelial markers (such as EpCAM) and have been known to evade capture by classical EpCAM-based CTC isolation systems [34,35,36]. However, in the context of PC, EMT CTCs are less well-described and there is evidence, in both humans and mouse models, to suggest that the expression of CK in these cells may remain intact, despite the gain of mesenchymal marker expression [37,38]. Furthermore, immunocytochemistry performed on cPSCs demonstrated that they stained for PSC-specific markers such as GFAP. Finally, in our scRNA-seq data, we found that none of the putative cPSCs identified expressed the luciferase transgene, thus strongly suggesting that these cells are distinct from the implanted luciferase-transfected cancer cells.

Characterisation of these rare circulating cells is challenging. We have presented single-cell transcriptomic characterisation data for cPSCs, which allowed the identification of potentially important cPSC genes involved in the metastatic process. Some of the most promising genes worthy of further investigations include insulin-like growth factor-binding protein 1 (*IGFBP1*), claudins and hyaluronan mediated motility receptor (*HMMR*).

IGFBP are a family of proteins which bind IGFs (insulin-like growth factors) to regulate their bioavailability and half-life. Insulin-like growth factor 1 (IGF-1) signalling has been known to influence the growth of PC cells in vitro, promote PC cell and PSC migration in vitro and is associated with poorer survival in patients with PC [39,40,41,42,43]. More recently, it has also been found to be an important part of tumour−stromal cross-talk [44]. 

Unlike *IGFBP1*, the role of claudins in PSC physiology has not previously been described. These form a component of tight junctions, classically associated with epithelial rather than mesenchymal cells. However, it should be noted that mesenchymal cells are not completely devoid of tight junctions or tight junction-like intercellular connections. For instance, junctional adhesion molecules (JAMs), components of tight junctions, are found to be expressed in fibroblasts and claudins have been found to be expressed in a variety of sarcomas [45,46]. Thus, PSC-expressed claudins may potentially play a role in the pathophysiology of PC [47]. Furthermore, this raises the possibility that intercellular junctions, such as tight junctions, may be another medium through which PSC−PC cross-talk can occur [48]. 

Hyaluronan mediated motility receptor (*HMMR*) is important in mesenchymal cell migration in wound repair, including fibroblast migration and differentiation into myofibroblasts [49]. In PC, strong expression of this protein by immunohistochemistry is associated with poorer survival in PC [50]. Surprisingly, its role in PSCs has not been well studied. However, HMMR (in combination with CD44) has been hypothesised to play an important role in cancer-associated fibroblast migration [51]. Most interestingly, fibroblasts transfected with *HMMR* injected subcutaneously in mice generated tumours which spontaneously metastasised to the lungs [52]. It is therefore conceivable that this may be an important factor in the generation (migration and intravasation) of cPSCs.

It is acknowledged that the transcriptomic analysis of cPSC physiology reported in this study will require protein- and functional-level validation. However, our findings provide novel insights to better understand how cPSCs support metastasis.

## 5. Implications and Conclusions

In summary, this paper describes the use of an effective adjuvant mouse model of pancreatic cancer developed in our laboratory [15] to demonstrate, for the first time, that combined HGF/c-Met inhibition is effective in retarding the rate of progression of disease after surgical resection of the primary tumour. There is now a strong argument to test this treatment strategy in a randomised controlled trial for PC patients.

We also describe, again for the first time, the identification and characterisation of circulating PSCs in the setting of pancreatic cancer. The trend for higher cPSC counts to be associated with recurrent disease after tumour resection, and their modulation with HGF/c-Met inhibition suggests that these cells play a significant role in the metastatic process. These cells highlight a potential gap in the current state of understanding of the pathogenesis of PC metastasis and may have wider implications for understanding the pathogenesis of cancer metastasis in general, given that PSCs in PC play a role analogous to that of cancer-associated fibroblasts in many other cancers [53]. 

## Figures and Tables

**Table 1:** Characteristics of the mice at the commencement of treatment.

Treatments	Ctrl	G	HiCi	G+HiCi	*p*-Value
	*n* (%) or mean ± SE				
*n* (%)	14 (23%)	15 (24%)	18 (29%)	15 (24%)	
***Resection Characteristics:***					
Resected tumour vol (mm^3^)	156 ± 22	217 ± 31	175 ± 20	205 ± 19	0.269
Macroscopically clear margins	14 (100%)	15 (100%)	18 (100%)	15 (100%)	
Extra-pancreatic involvement	3 (21%)	4 (27%)	8 (44%)	4 (27%)	0.560
***Tumour Burden at Commencement:***					
Ventral radiant flux at treatment start (base 10 log units) (10^x^ p/s)	5.8 ± 0.22	6.0 ± 0.27	5.9 ± 0.19	6.0 ± 0.31	0.925

**Figure A14:**
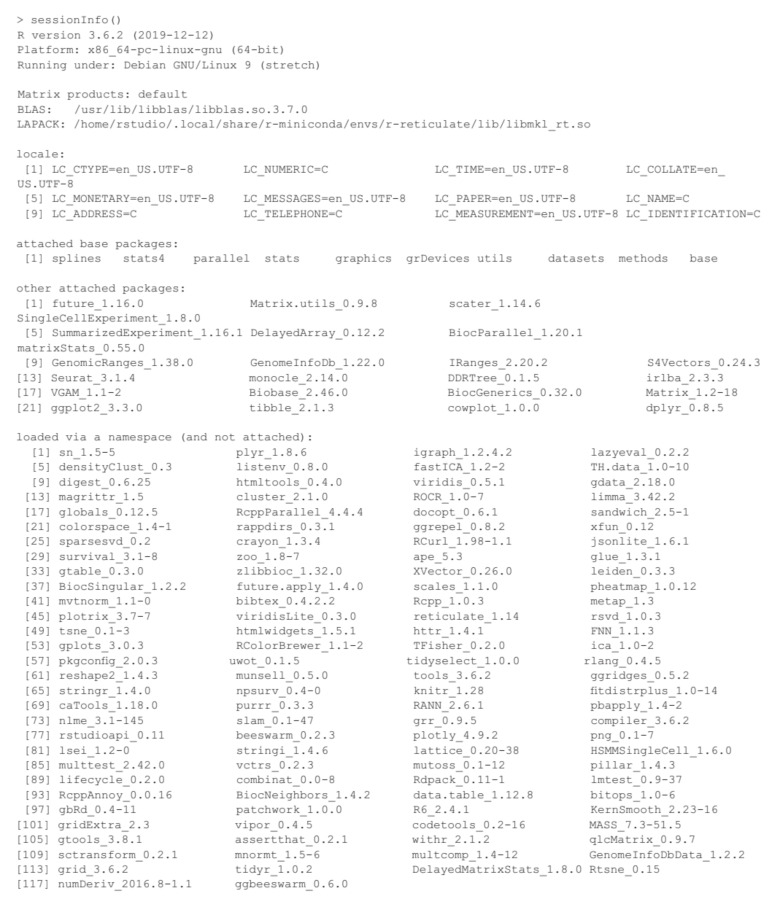
Details of R session and loaded packages.

## Data Availability

The data presented in this study are openly available in GEO (accession GSE175837).

## Appendix A

This provides a detailed description of the experimental methods which could not be included in the main manuscript due to length limitations.

### Appendix A.1. Orthotopic Mouse Models

Two orthotopic mouse models were used in this study: Model 1 (adjuvant treat-ment model) and Model 2 (cPSC characterisation model). The aim of Model 1 was to test the effectiveness of HGF/c-Met inhibition treatment in the post-resection setting. This model thus involved tumour implantation, tumour resection, drug treatments and disease analysis (tumour burden, circulating cells, immunohistochemistry). Model 2, in contrast, was an observational model designed to identify and characterise circulating PSCs by single cell RNA-sequencing (scRNA-seq). Thus, this model involved only tumour implantation, the passage of time for tumour progression, then collection of blood and tissue for scRNA-seq analysis. Summary flowcharts for each model are shown in Figure A1 and Figure A2.

### Appendix A.2. Tumour Implantation

All three models were based upon the same basic orthotopic co-injection model described previously [14]. Briefly, athymic nude mice (BALB/c-*Fox1*^nu^/Ausb), aged 8−10 weeks and weighing 16−18 g, were anaesthetised and the pancreas was exteriorised through a left cranial quadrant abdominal incision. The pancreas was injected with a mixture of 10^6^ luciferase-tagged AsPC-1 cells (kindly provided by Professor Takashi Murakami, Saitama University, Saitama, Japan) and 10^6^ cancer-associated human PSCs (Pancreatic Research Group PSC bank) in 50 μL phosphate-buffered saline (PBS) with a 29 G insulin needle. The spleen and pancreatic tail were then returned and abdomen was closed. Mice were monitored for 4 h postoperatively. Postoperative monitoring was performed daily, and body weights measured twice a week. Humane end-points included loss of body weight > 20%, features of untreatable distress, tumour size > 1 cm^3^. Mortality from tumour progression was not considered as an acceptable humane endpoint, therefore, no survival analysis was possible.

In the adjuvant treatment model (Model 1), additional precautions were taken during tumour implantation to prevent inadvertent peritoneal seeding (which would subsequently preclude resection with curative intent of the cancer). These precautions included: the use of an injection device which allows slow injection of the cell suspension, and the use of a purse-string gauze to absorb any inadvertent spillage during injection. Details of these modifications are described previously [15].

### Appendix A.3. Tumour Resection

Open distal pancreatectomy and splenectomy was only performed in the adjuvant treatment model (Model 1). The period between implantation and resection was three weeks, determined empirically to represent the latest time point for resection while maintaining tumour resectability. Bioluminescence imaging (described below) was performed the day prior to planned resection to exclude mice with clearly disseminated disease.

Figure A3 illustrates the tumour resection procedure. The mice were anaesthetised and the previous incision site re-incised. The old suture line was excised (and fixed in formalin), in case of local suture recurrence. After examining the abdominal cavity for evidence of metastasis, the spleen and distal pancreas were exteriorised. A plane dorsal to the body of the pancreas was bluntly dissected and the pancreas was ligated in continuity with a titanium ligation clip, at least 5 mm from the tumour. The lienogastric vessels were cauterised on the deep surface. The specimen was removed, and the abdominal wall closed. Postoperative monitoring and humane endpoints were identical to those described above. Details of the resection procedure has been described previously [15].

### Appendix A.4. Treatments

In Model 1, mice were treated with gemcitabine and/or combination rilotumumab and Compound A (c-Met small molecule inhibitor) in a 2 × 2 factorial design. The four groups were therefore:

- **Control** (**Ctrl**)**: IgG,** 300 μg/mouse, by intraperitoneal (i.p.) injection daily (Amgen Inc., Thousand Oaks, CA, USA) and soybean oil, 10 μL/g by daily oral gavage (Sigma-Aldrich Pty Ltd., Castle Hill, NSW, Australia);
- **Gemcitabine** (**G**): Gemcitabine, 75 mg/kg i.p. daily (Hospira, Mulgrave, VIC, Australia) alone;
- **HGF/c-Met inhibition** (**HiCi**)**:** combination of rilotumumab, 300 μg/mouse i.p. daily (Amgen Inc.) and Compound A, suspended in soybean oil, 60 mg/kg by daily oral gavage (Amgen Inc.)
- **Combination of Gemcitabine and HGF/c-Met inhibition (G+HiCi)**: Gemcitabine, rilotumumab and Compound A.

Treatments were commenced one-week post-resection, and continued for eight weeks (Figure A1).

Model 2, used for transcriptomic analysis, involved no treatments.

### Appendix A.5. In Vivo Bioluminescence Imaging

For the adjuvant treatment model (Model 2), bioluminescence imaging was performed weekly, after implantation of the tumour, until the end of the treatment period to non-invasively track tumour progression.

D-luciferin (15 mg/mL. PerkinElmer Inc., Waltham, MA, USA) was administered by intraperitoneal injection and anaesthesia was induced and maintained with isoflurane (3−4% with oxygen. Zoetis Australia Pty Ltd., Rhodes, NSW, Australia). Imaging was performed using IVIS Lumina II (Caliper Life Sciences, Hopkinton, MA, USA) 18−26 min after injection. The timing of imaging was determined empirically using a luciferin kinetic curve. Image data analysis was performed using Living Image version 4.5.5 (64-bit) for Windows (Caliper Life Sciences). Total tumour burden was semi-quantitatively estimated by the ventral bioluminescent flux of the mouse, adjusted for the background radiance, using the formula *F_c_ = F_m_ − F_b_ (A_m_/A_b_)*. Where *F_c_* is the corrected radiant flux, *F_m_* is the measured radiant flux of the mouse ROI, *F_b_* is the radiant flux from the background ROI, and *A_m_* and *A_b_* are the areas of the mouse and background ROIs, respectively. The regions of interest representing the ventral surface of the animal and of the background were drawn in manually.

### Appendix A.6. Necropsy and Blood Sample Collection

At the end of the study period, mice were euthanased by CO_2_ exposure and cervical dislocation. The peritoneal cavity was entered with a cruciate incision. The abdominal contents were reflected to the left and the portal vein punctured with a 29 G needle on a 0.5 mL insulin syringe for aspiration of a target volume of 20 mL/kg of blood. Blood samples were immediately placed into a CellSave tube (for Model 1; Menarini-Silicon Biosystems) or an EDTA tube (for Model 2; Interpath Service Pty Ltd., West Heidelberg, Victoria, Australia) and was mixed well by inversion.

The abdominal and chest cavities were then examined for evidence of metastasis, which was recorded on a standard chart. The primary tumour was excised and the tumour volume estimated according the formula (½ × length × width × height) [16].

### Appendix A.7. Enrichment of Circulating Tumour Cells and Circulating Pancreatic Stellate Cells

Circulating cell enrichment in Model 1 was performed using a filtration-based system, CellSieve platform (Creatv Micro Tech, Rockville, MD, USA), according to the manufacturer’s instructions. Briefly, the sample was pre-fixed using the provided prefixation buffer, incubated, then loaded into the input syringe. A syringe driver was used to aspirate the sample through the filter at 2 mL/min. The filter membrane was then washed and immunocytochemistry was performed directly on cells bound to the membrane.

Circulating cell enrichment in Model 2 was performed using a negative immunomagnetic technique. After red cell lysis, the sample was incubated with the Mouse Cell Depletion Microbead cocktail (Miltenyi Biotec, Macquarie Park, NSW, Australia) and processed using the AutoMACS Pro cell separator (Miltenyi Biotec) using the sensitive depletion (“depletes”) protocol. The volume and concentration of the cells were then adjusted to achieve the target necessary for the 10× Genomics Chromium platform (10× Genomics, Pleasanton, CA, USA).

### Appendix A.8. Identification of Cells on Immunocytochemistry

Immunocytochemistry of the enriched cells was performed directly on the filtration membrane following filtration enrichment. Cells were fixed with 4% paraformaldehyde, permeabilised with 0.3% Triton x-100 (Sigma-Aldrich Pty Ltd., Castle Hill, NSW, Australia) and blocked with normal donkey serum (Jackson ImmunoResearch Laboratories Inc., West Grove, PA, USA). They were then incubated with rabbit polyclonal anti-CD45 primary antibody (1:200. Abcam plc, Cambridge, UK), donkey anti-rabbit IgG conjugated with AlexaFluor 647 (1:800. Jackson ImmunoResearch Laboratoraties Inc.). After further blocking with normal mouse serum (Jackson ImmunoResearch Laboratories Inc.), the cells were incubated with monoclonal mouse anti-human α-smooth muscle actin (clone 1A4) antibody conjugated with Cyanine 3 (1:150. Sigma-Aldrich Pty Ltd.) and monoclonal mouse anti-human pan-cytokeratin (clone C-11) antibody conjugated with fluorescein isothiocyanate (FITC) (1:450. Sigma-Aldrich Pty Ltd.). This was mounted and nuclear stained with Fluoromount-G with DAPI (Life Technologies Corporation, Tullamarine, VIC, Australia).

Selected filter samples had their fluorescent signals quenched with sodium borohydride and re-stained with further antibodies based on the method of Adams et al. [17] The filter membrane was washed with PBS, then incubated in sodium borohydride solution (1 mg/mL. Sigma-Aldrich Pty Ltd.), then 100mM tris solution. After washing, post-quench images were taken to verify successful quenching of the original fluorescent signal. Re-staining was then performed by 5% bovine serum albumin (Roche Diagnostics, Indianapolis, IN, USA), followed by incubation with monoclonal mouse anti-human glial fibrillary acidic protein (clone GA5, conjugated with Alexafluor 488) (1:100. Novus Biologicals USA, Centennial, CO, USA) and monoclonal rabbit anti-human desmin (clone Y66, conjugated with Alexafluor 594) (1:100. Abcam plc). Mounting was again with Fluoromount-G with DAPI.

Widefield microscopy was performed with Olympus IX71 with an automated stage. Random low powered fields (10× main objective) were sampled. Four channel confocal microscopy images were also acquired using a Zeiss LSM800 microscope for selected cells.

Cells were enumerated using a semi-automated technique using ImageJ version 1.52i (National Institutes of Health, USA). The process is summarised in Figure A4. In brief, individual image channels underwent rolling ball background subtraction (50 px). Thresholding was performed manually. Cells, determined by DAPI nuclear staining, were classified into positive or negative staining for the other markers (CD45, CK and α-SMA) using the assigned thresholds. Quality control was performed by determining the proportion of cells with indeterminate identity (overlapped staining). Samples with less than 10% of cells with overlapped staining were passed while those with more than 10% had their thresholds further adjusted. This adjustment was guided by the pattern of overlap as represented by a heatmap. This was done iteratively as required. The accuracy of this semi-automated technique was validated by comparing with manual enumeration in n = 38 samples. The Bland−Altman plot demonstrated good agreement across a wide range of CTC counts (data not shown).

Cells thus classified were counted automatically. cPSCs were further reviewed manually to confirm these do not represent misidentified debris. CTCs were sampled (20 per blood collection) for a similar quality check.

Cell clusters were identified manually after generation of a binary image map using cell classifications above (Figure A4c). Cells were defined as “clustered” if 2 or more cells were either in direct contact or within 1 μm of each other.

The cPSCs identified above were manually classified into 4 different morphological patterns by a single operator. The measurements of cell size, circularity and staining intensity were performed by the use of the *magic wand* function of ImageJ to establish a region of interest (ROI) (Figure A4d). Measurements of these ROIs were then made.

### Appendix A.9. Immunohistochemistry

Immunohistochemistry of paraffin-embedded sections of local recurrent tumour of the adjuvant treatment model (Model 1) was performed. The sections were deparaffinised by xylenes and graded ethanol. Antigen retrieval was performed using either Tris or citrate antigen retrieval buffers. Primary antibodies used included rabbit polyclonal anti-DCLK1 (1:800. Sigma-Aldrich Pty Ltd., Castle Hill, NSW, Australia), mouse monoclonal anti-human cytokeratin (clones AE1/AE3) (1:100. Agilent Technologies Pathology, Mulgrave, VIC, Australia), rabbit polyclonal anti-Ki67 (1:300. Abcam plc, Cambridge, UK), rabbit polyclonal anti-CD31 (1:50. Abcam plc). This was followed by incubation with relevant secondary antibodies (Goat anti-rabbit immunoglobulin [Agilent Technologies Pathology] and goat anti-mouse immunoglobulin [Agilent Technologies Pathology]) and DAB visualisation. TUNEL (terminal deoxynucleotidyl transferase dUTP nick-end labelling) staining was performed using In-situ Cell Death Detection Kit (Roche Diagnostics, Indianapolis, IN, USA). Morphometric analysis and cell counting was performed using ImageJ version 1.52i.

### Appendix A.10. Single Cell RNA Sequencing

Cell partitioning was performed using the 10× Genomics Chromium platform (10× Genomics, Pleasanton, CA, USA) according to the manufacturer’s instructions. Reverse transcription, amplification and expression libraries were constructed with reagents from the Chromium Single Cell 3’ Reagent Kit version 3 (10× Genomics). Sequencing was performed at the Ramaciotti Centre for Genomics (UNSW) using the Illumina NovaSeq 6000 platform (Illumina, Inc., San Diego, CA, USA) with the SP 100 cycle flow cell with read lengths 28, 8, 91 bp.

### Appendix A.11. Bioinformatics

Generation of expression matrices from base call files was performed using the CellRanger pipeline, version 3.1.0 (10× Genomics). A custom combined reference genome was compiled for sequence alignment based on GRCh38 human assembly (Ensemble release 93), GRCm38 mouse assembly (release 93), modified firefly (*Photinus pyralis*) luciferase transgene, *luc+*, from the pGL3 Basic plasmid (Promega Corporation, Madison, WI, USA). The latter included the SV40 sequence upstream of the polyadenylation signal. The basic sequencing and alignment metrics are shown in Table A1.

Processing of the expression matrix data was performed using Seurat (version 3.1.4) [18], and Monocle (version 2.14.0) [19] packages in R (version 3.6.2). The details of the software and packages used in this analysis are listed in the sessionInfo() (Figure A14). Cells were first filtered by “quality”: gene counts, RNA molecule count and mitochondrial gene content. Gene expression data was then normalised using the LogNormalize method, with a scale factor of 10,000. Variable genes (2000 genes) were then identified using a method based on variance stabilising transformation [18], and the data were scaled and centred.

Linear and non-linear dimensional reduction was performed and visualised by principal component analysis and UMAP/t-SNE (uniform manifold approximation and projection/t-stochastic neighbour embedding) plots, respectively. For cell identification, unsupervised graph-based clustering (as implemented in Seurat) was used to determine groups of cells with similar expression profiles. Clusters were then identified based on a combination of differential expression and canonical marker expression.

**Table A1:** Basic sequencing and alignment metrics for the four samples.

	Mouse Primary *(MsPrim)*	Mouse Secondary *(MsSec)*	Mouse Blood *(MsBlood)*	Cultured Cells *(Cult)*
Reference genome used for alignment	Human/*luc+*+ Mouse	Human/*luc+*+ Mouse	Human/*luc+*+ Mouse	Human/*luc+*
				
*Cell metrics*				
Estimated number of cells	1952	3850	6764	5131
Estimated human cells	260	84	1513	5131
Estimated mouse cells	1696	3767	5262	-
Fraction GEMs with >1 cell (%) (95%CI) *	2.3 (0.9−3.8)	1.3 (0.0−3.5)	0.8 (0.5−1.2)	-
*Sequencing depth metrics*				
Mean reads per cell	131,113	68,694	35,043	48,214
Median genes per cell (human)	2449	664	94	3393
Median genes per cell (mouse)	1662	1752	1028	-
Sequence saturation	83.6%	72.5%	71.1%	27.9%
				
*Sequencing metrics*				
Q30 bases in RNA read ^†^	93.4%	93.6%	93.6%	94.1%
				
*Alignment metrics*				
Reads mapped confidently to genome	89.3%	88.0%	84.4%	93.8%
Human	23.7%	8.7%	0.3%	93.8%
Mouse	65.6%	79.2%	84.1%	-
Reads mapped confidently to transcriptome	55.3%	56.1%	55.1%	69.9%
Human	14.1%	5.5%	0.2%	93.8%
Mouse	41.3%	50.5%	54.9%	-
				
Human cells left after filtration	100	40	75	2791

Notes: * This estimates the fraction of multiplets in the sample. It is calculated from the fraction of GEMs with significant number of reads mapped to both human and mouse genomes, which is then extrapolated to all GEMs. This is therefore not estimable when only the human reference genome was used (hence the absence of a value in the cultured cell sample). ^†^ Fraction of RNA read bases with Q-score ≥ 30, excluding very low quality/no-call (Q ≤ 2) bases from the denominator.

#### Appendix A.11.1. Circulating PSC Identification

As only human PSCs and AsPC-1 cells were implanted, circulating PSCs were identifiable by combining three pieces of information: human versus mouse identity, PSCs versus PC cell identity, and the tissue compartment of origin (primary tumour versus secondary versus blood specimens). The general strategy for circulating rare cell identification is summarised in Figure 5a.

Two methods were used to distinguish human from mouse cells. The first technique was based on the relative proportions of transcripts (unique molecular identifiers [UMI] counts) aligned to either mouse or human reference genomes assigned to a barcode. The second technique relied on graph-based clustering (as above) to distinguish cell species based on gene expression. To distinguish cells based on gene expression, only a subset of genes where there was one-to-one mouse−human orthology was used for analysis. This list was downloaded from Ensembl (Release 99). Both mouse and human genes within this subset were converted to the human gene name. Normalisation, scaling, dimensional reduction, clustering, and identification then proceeded as described above.

Three methods were used to distinguish PSCs versus PC cells (Figure A5). The first two methods involved combining the human cells from the primary tumour, blood and metastasis samples with the culture sample into an integrated dataset using the same anchor-based technique [54]. This allowed the cells in the mouse model samples to be mapped to the cultured cell sample. Based on the resulting UMAP plot co-ordinates, the cells were then identified as either PC cells or PSCs (approach 1). This classification was then cross-checked with cell type classification based on unsupervised clustering (approach 2).

To confirm the robustness of this cell type classification, a third classification approach avoided data integration. This strategy first identified the most important genes which distinguished between PC cells and PSCs in the culture sample, then applied these genes to differentiate the mouse model samples, as described below. Cells in the culture (Cult) sample were classified into either PC cells or PSCs by unsupervised cell clustering using canonical markers (*KRT18, luc+* for AsPC-1 and *ACTA2*, *COL1A1*, *SPARC* for PSCs). Differential gene expression analysis was then performed to identify the top 500 markers for each cell type (as ranked by their specificity score [55]). These 1000 marker genes were then used to perform unsupervised cell clustering (t-SNE-based [56]) to distinguish between CTCs and cPSCs. In contrast to the first two approaches, this was performed with a different software package (Monocle 2) to further reduce shared assumptions with the first approaches.

#### Appendix A.11.2. Differential Expression Analysis

Differential expression analysis was then performed on the original transcript counts to compare the PSCs and CTSs. Due to the small numbers of cPSCs and CTCs identified (total *n* = 78), expression of *luc+* gene in AsPC-1 cells was compared to that of cPSCs across all specimens (cPSCs should not express the *luc+* gene). As this is the only comparison of significance between these cell types and was determined a priori, the unadjusted *p*-value was reported.

#### Appendix A.11.3. Trajectory Analysis

Single cell trajectories were constructed using the reversed graph embedding technique implemented by Monocle 2 [19]. Trajectories were constructed for PSCs. The cells from the different samples were first integrated using the Seurat package, as described above. The batch-corrected expression for the top 2000 differentially expressed genes was then used to calculate the single cell trajectory using Monocle 2. The limb of the trajectory with the highest proportion of primary cancer cells was assigned the earliest pseudo-time to determine the origin of the trajectory.

Differential expression analysis (using the non-corrected transcript counts) was then performed using the Monocle 2 package to evaluate genes whose expression changes over the trajectory (i.e., over pseudo-time) and those which are differentially expressed by cells passing down different paths at a branch point.

These differentially expressed genes were then analysed as a protein network using the STRING database version 11.0 (https://string-db.org) [57]. This is a database of known and predicted protein-protein interactions. Pathway enrichment analysis was also performed within STRING, and the results of this analysis as related to biological processes were inferred based on KEGG (Kyoto Encyclopaedia of Genes and Genomes) pathways [58], Reactome pathways [59], and GO (Gene Ontology) [60]. In addition to analysing the network, individual genes were also screened for potential biologically relevant actions based on data in the literature.

### Appendix A.12. Non-Bioinformatics Statistical Analysis

Descriptive statistics for parametric, non-parametric and categorical variables were presented as mean ± SE, median (interquartile range, IQR) and *n* (%) respectively. Raw circulating rare cell counts were adjusted to account for the sample collection volume and were reported as counts per 200 μL of sampled blood. These circulating rare cell counts were, for most analyses, logarithmically transformed prior to statistical testing. Basic inferential tests, including ANOVA/*t*-test, Kruskall Wallis test and Fisher’s exact/chi-squared tests, were first performed to explore the relationship between variables. If the global test demonstrated a near-significant result (*p*-value < 0.1), then post-hoc Tukey’s multiple comparison test was performed.

For regression modelling, multilevel mixed-effects modelling was performed. For Model 2, only *set*-level clustering was taken into account in the model. *Sets* were defined as mice which were implanted (and possibly resected), housed, treated and euthanased together, and their blood samples were processed, stained, and imaged together. The dependent variables of interest (for instance, treatments) were considered as fixed effects. The added value of the multilevel model over a simple multivariate regression model was assessed by performing a likelihood ratio test—the simpler model was generally favoured if the test failed to reject the null hypothesis (*p*-value > 0.05).

Treatments were considered as two independent treatment groups due to the 2 × 2 factorial pattern. First-order interactions were included in initial regression models, but these was dropped if the corresponding *p*-value exceeded 0.1 or if only one of the treatments was significant. Other independent variables were included in the model if these were found to be significant or near significant (*p* < 0.15) on univariate analysis. Missing data, which were rare and due to technical issues related to measurement rather than to biology, were treated as being Missing at Random (MAR).

As circulating rare cell counts has a skewed distribution and commonly included zero counts, modelling of circulating rare cell counts was performed with Poisson regression. Robust standard errors (estimated using the Huber/White/sandwich linearised estimator) were reported [61,62].

For longitudinal analysis of tumour burden progression, as estimated by the total radiant flux from the ventral surface of the animal, multilevel mixed-effects log-linear regression model was used. Animal-level variation in tumour burden was allowed for by a random intercept term. Fixed effect independent variables included time (postoperative days), variables of interest (such as treatments) and interaction terms between times and these variables. The coefficient of the time variable represents the slope of the baseline case while the interaction terms (with time) represent changes to this slope by these other variables.

Tumour progression was also analysed by assessing the probability of progressive disease. Animals whose ventral radiant flux increased over time significantly (both statistically and quantitatively) were considered to have progressive disease. In this case, each individual’s disease progression trajectory was quantified by linear regression. Mice with statistically significant positive slopes exceeding a threshold set a priori (to reflect a clinically significant change) were considered to have progressive disease. This threshold was set at 10-fold change in ventral radiant flux over 50 days (this duration approximated the treatment period of 8 weeks). Logistic regression analysis was then performed to assess the predictors for this classification.

All non-bioinformatics statistical analysis and data visualisation were performed using either GraphPad Prism version 8.3.0 for Windows (GraphPad Software LLC, San Diego, CA, USA) or Stata 15.1 IC for Windows (StataCorp LLC, College Station, TX, USA).

## Appendix B

This Appendix contains the full specifications of statistical models represented graphically in Figure 1, Figure 2 and Figure 3.

**Table A2:** Model specification for Figure 1a: mixed-effects model for longitudinal analysis of tumour burden for all mice. (**a**) Multivariate model of the ventral radiant flux of mice. The coefficients represent the log10 value of the n-fold change associated with one unit change in the covariate. To make this model easier to interpret, the initial flux value was *centred* around 10^6^ p/s (that is, 10^6^ is 0, 10^7^ is 1, etc.). (**b**) As the model may be confusing for the reader, this table shows the predicted rates of progression under various conditions (according to this model). The rate of progression is in this table expressed as *n*-fold changes of ventral radiant flux per week.

(**a**) Multivariate model for disease progression, as measured by ventral radiant flux, over time.				
	**Coefficient** (**log 10 Scale**)	**95% CI**	**z**	***p*-Value**
*Baseline slope:*				
Treatment weeks (per week)	0.130	0.101 to 0.159	8.79	< 0.001
*Factors affecting slope:*				
Gemcitabine (G)	−0.050	−0.089 to −0.0113	−2.53	0.011
Rilotumumab + Compound A (HiCi)	−0.069	−0.106 to −0.031	−3.61	< 0.001
G × AR interaction	0.064	0.0102 to 0.117	2.33	0.02
Initial flux (per 10-fold change)	0.070	0.053 to 0.087	8.14	<0.001
*Other model terms:*				
Initial flux (per 10-fold change)	0.98	0.82 to 1.1	12.3	< 0.001
Intercept	6.0	5.9 to 6.2	78.8	< 0.001
*Random effects (intercept):*				
Variance	0.257	0.176 to 0.376		
				
(**b**) Rate of progression of disease as predicted by the multivariate model above				
	***n*** **-Fold Change per Week**		**95% CI**	
Baseline (initial flux 10^6^ p/s, Ctrl)	1.35		1.26 to 1.44	
*Treatment groups:*				
Rilotumumab + Compound A (HiCi)	1.15		1.09 to 1.22	
Gemcitabine alone (G)	1.20		1.13 to 1.28	
G+AR	1.19		1.11 to 1.27	
*Initial tumour burden:*				
Flux of 10⁷ p/s, Ctrl group	1.59		1.46 to 1.72	
Flux of 10⁸ p/s, Ctrl group	1.87		1.67 to 2.08	

**Table A3:** Model specification for Figure 1c: Logistic regression model predicting probability of progressive disease. Progressive disease was defined by a statistically and clinically significant increase in the disease burden (see detailed definition in the text). The odds ratio represents the change in the odds of progressive disease when the relevant variable is true (compared to the baseline case). The intercept for this model represents the baseline odds of progressive disease.

	Odds Ratio	95% CI	z	*p*-Value
Gemcitabine alone (G)	0.80	0.172 to 3.68	−0.29	0.771
Rilotumumab + Compound A (HiCi)	0.15	0.0250 to 0.86	−2.13	0.033
Initial flux (per 10-fold change from 10^6^ p/s)	4.7	1.95 to 11.2	3.45	0.001
Baseline odds (Ctrl, initial flux of 10^6^ p/s)	0.45	0.136 to 1.50	−1.30	0.195

**Table A4:** Model specification for Figure 1d: mixed-effects model for longitudinal analysis of tumour burden for mice with recurrent disease. (**a**) The Table shows the statistical model of the effect of treat-ments and initial tumour burden on the rate of progression of disease in this subgroup. The co-efficient for treatment weeks represents the baseline slope, while the coefficients for the interac-tion terms with treatment weeks (labelled in the table as “factors affecting slope”) represent fac-tors which change the slope of the disease burden curve. The units for these coefficients are log10 n-fold change of radiant flux per week. The baseline condition represents mice with an initial flux of 10⁶ p/s in the control group, although the baseline for a given animal is assumed to vary (random intercepts model). (**b**) To assist the reader in interpreting this model, this Table shows the rate of progression of disease, predicted by the model, of mice under various conditions. The rate of change is expressed as an n-fold change in the radiant flux per week.

(**a**) Multivariate model for disease progression, as measured by ventral radiant flux, over time				
	**Coefficient**	**95% CI**	**z**	***p*-Value**
*Baseline slope:*				
Treatment weeks (per week)	0.199	0.153 to 0.246	8.39	<0.001
				
*Factors affecting slope:*				
Gemcitabine (G)	−0.051	−0.119 to 0.0179	−1.45	0.148
Rilotumumab + Compound A (HiCi)	−0.086	−0.152 to −0.019	−2.54	0.011
G×HiCi interaction	0.092	−0.0077 to 0.192	1.81	0.070
Initial flux (per 10-fold change)	0.0242	−0.0035 to 0.052	1.71	0.087
				
*Other model terms:*				
Initial flux (per 10-fold change)	1.01	0.76 to 1.26	7.91	<0.001
Intercept (Baseline)	6.06	5.8 to 6.4	37.9	<0.001
				
*Random effects (intercept):*				
Variance	0.403	0.227 to 0.72		
(**b**) Rate of progression of disease as predicted by the multivariate model above				
	***n*-Fold Change per Week Estimated by Model**		**95% CI**	
Baseline (mouse with 10^6^ p/s initial radiant flux, control group)	1.58		1.42 to 1.76	
		
*Treatment groups:*				
Rilotumumab + Compound A (HiCi)	1.30		1.16 to 1.46	
Gemcitabine alone (G)	1.41		1.25 to 1.59	
G+HiCi	1.43		1.22 to 1.68	

**Table A5:** Model specification for Figure 2b: Linear regression model of Ki67 positivity versus treatments.

	*n*-Fold Change	95% CI	t	*p*-Value
Gemcitabine (G)	5.6	1.17 to 27.0	2.4	0.034
Rilotumumab + Compound A (HiCi)	0.47	0.100 to 2.18	−1.08	0.302
Intercept (baseline rate of Ki67+)	104	24.6 to 437	6.77	<0.001

Note: interaction term was dropped as it was not statistically significant.

**Table A6:** Model specification for Figure 2c: Linear regression model of CD31 positive area as a percentage of tumour cross sectional area.

	Coefficient (Change in % CD31+)	95% CI	t	*p*-Value
Gemcitabine (G)	0.50	0.0036 to 1.0	2.22	0.049
Rilotumumab + Compound A (HiCi)	−0.54	−1.0 to −0.041	−2.38	0.036
				
Intercept (baseline percent CD31+)	0.76	0.31 to 1.2	3.74	0.003

**Table A7:** Model specification for Figure 4d: Poisson regression model of CTC counts versus treatments. This model assesses the effects of treatments on CTC counts, taking into account the last measured tumour burden (assessed by bioluminescence imaging). Note: The interaction term was dropped as it was not statistically significant.

	*n*-Fold Change	95% CI	z	*p*-Value
Gemcitabine (G)	0.370	0.162 to 0.844	−2.36	0.018
Compound A and rilotumumab (HiCi)	0.396	0.172 to 0.91	−2.18	0.029
Last radiant flux > 10^6^ p/s	3.10	1.42 to 6.8	2.83	0.005
Intercept (Baseline CTC count)	4.9	2.88 to 8.5	5.81	<0.001

**Table A8:** Model specification for Figure 4e: Poisson regression model of portal vein cPSC counts versus treatments. This model assesses the effects of treatments on cPSC counts, taking into account the tumour burden at the end of the experiment (as measured by bioluminescence flux). Note: The interaction term was dropped as it was not statistically significant.

	*n*-Fold Change	95% CI	z	*p*-Value
Gemcitabine (G)	0.58	0.191 to 1.76	−0.96	0.336
Compound A and rilotumumab (HiCi)	0.283	0.100 to 0.80	−2.29	0.017
Recurrent disease	2.43	0.86 to 6.9	1.67	0.096
Intercept (Baseline cPSC count)	0.57	0.222 to 1.44	−1.19	0.234

## Appendix C

This appendix contains additional supporting materials.

**Table A9:** Enriched pathways amongst upregulated PSC genes over pseudo-time. This Table summarises the pathways identified as being over-represented in the protein network presented in Figure A12. The pathways explored included Gene Ontology (GO) biological processes, KEGG pathways and Reactome pathways. Abbreviations: Bkg, Background (number of genes in the pathway). FDR, false discovery rate.

Pathway Accession	Description	Genes	Bkg	FDR
*GO Process*				
GO:0045669	Positive regulation of osteoblast differentiation	CEBPB, JUND	58	0.0020
GO:0009719	Response to endogenous stimulus	CLDN4, JUND, CEBPB, IGFBP1, PMEPA1	1353	0.0345
*KEGG*				
hsa04657	IL-17 signalling pathway	CEBPB, JUND	92	0.0100
*Reactome*				
HSA-8957275	Post-translational protein phosphorylation	CYR61, IGFBP1	106	0.0382
HSA-381426	Regulation of IGF transport and uptake by IGFBPs	CYR61, IGFBP1	123	0.0382
